# Cryo-thermal therapy induces macrophage polarization for durable anti-tumor immunity

**DOI:** 10.1038/s41419-019-1459-7

**Published:** 2019-03-04

**Authors:** Kun He, Shengguo Jia, Yue Lou, Ping Liu, Lisa X. Xu

**Affiliations:** School of Biomedical Engineering and Med-X Research Institute, Shanghai Jiao Tong University, Shanghai, China

## Abstract

Many cancer therapies are being developed for the induction of durable anti-tumor immunity, especially for malignant tumors. The activation of antigen-presenting cells (APCs), including macrophages and dendritic cells (DCs), can bridge innate and adaptive immune responses against tumors. However, APCs have an immunosuppressive phenotype and reversing it for effective tumor-specific antigen presenting is critical in developing new cancer treatment strategies. We previously developed a novel cryo-thermal therapy to treat malignant melanoma in a mouse model; long-term survival and durable anti-tumor immunity were achieved, but the mechanism involved was unclear. This study revealed cryo-thermal therapy-induced macrophage polarization to the M1 phenotype and modulated the phenotypic and functional maturation of DCs with high expression of co-stimulatory molecules, increased pro-inflammatory cytokine production, and downregulated immuno-inhibitory molecule expression. Further, we observed CD4^+^ T-cell differentiation into Th1 and cytotoxic T-cell sub-lineages and generation of cytotoxic CD8^+^ T cells, in which M1 macrophage polarization had a direct, important role. The results indicated that cryo-thermal-induced macrophage polarization to the M1 phenotype was essential to mediate durable anti-tumor immunity, leading to long-term survival. Thus, cryo-thermal therapy is a promising strategy to reshape host immunosuppression, trigger persistent memory immunity for tumor eradication, and inhibit metastasis in the long term.

## Introduction

Metastasis accounts for the majority of cancer-related deaths. Conventional tumor therapy such as chemotherapy and radiotherapy alone can partially cure patients with advanced cancer, but their effectiveness for distant metastasis is limited^1^. Although immunotherapy holds great promise for cancer treatment, stimulation of an immune response to completely prevent distant metastasis is still far from being satisfactory. Cancer cells exploit multiple mechanisms to create an immunosuppressive environment^2–6^. The therapeutic effect of immunotherapy can be greatly impaired by an immunosuppressive environment^1,7,8^. Therefore, reversing immunosuppression and inducing durable anti-tumor immunity are essential in cancer therapy.

The natural antigen-presenting cells (APCs), such as macrophages and dendritic cells (DCs), are capable of bridging innate and adaptive anti-tumor immune responses^9^. However, these APCs can be induced to the immunosuppressive phenotype by other immunosuppressive cells and pro-tumor factors/molecules, having a pivotal role in tumor metastasis^10–12^. Tumor-associated macrophages (TAMs) can be divided into two subtypes, M1 and M2^11,13^. M2 macrophages secreting a high level of interleukin (IL)-10 and a low level of IL-12 can suppress T-cell activation and proliferation^13^. In contrast, macrophages can also be differentiated into activated macrophages (M1) producing a large amount of pro-inflammatory cytokines, with high capacity for antigen presentation in an ideal environment^14^. Moreover, recent studies indicate that M1 macrophages can modulate T-cell proliferation, differentiation, and formation of long-term anti-tumor immunity, suggesting that strong anti-tumor immunity requires the polarization of macrophages toward the M1 phenotype^11^. Similar to TAMs, there are three DC populations (termed as immature DCs, semi-mature DCs, and fully mature DCs) based on phenotype and function^15^. Only fully mature DCs are able to stimulate T cells, hereby increasing T-cell proliferation and secretion of interferon (IFN)-γ^16^. Recent studies indicate that systemic DC activation modulates immunosuppression and shapes long-lived memory T cells, suggesting that a strong adaptive immune response against tumors requires full DC maturation^17^. Hence, the plasticity of these immune cells would provide an opportunity for exploring novel treatments.

In our previous study, we developed a novel cryo-thermal therapy through applying local rapid cooling followed by rapid heating of a tumor^18–24^. Cryo-thermal therapy induced complete regression of implanted melanoma and prolonged long-term survival. The treatment markedly promoted differentiation of CD4^+^ T cells, which contributed to the induction of a durable specific memory immune response^25^. However, the mechanisms involved in inducing durable systemic memory anti-tumor immunity remain unclear.

We proposed that cryo-thermal therapy activated the tolerant innate immune system, triggered polarization of TAMs, promoted systemic DC maturation, and finally shaped long-term memory T cells. In this study, a murine B16 melanoma model was used. We found that cryo-thermal-induced macrophage polarization toward the M1 phenotype was exclusively responsible for subsequent DC maturation, differentiation of CD4^+^ T cells to Th1 and cytotoxic T cells (CTLs), and generation of cytotoxic CD8^+^ T cells, which in turn was crucial for mediating cryo-thermal-induced long-term anti-tumor memory. The current study suggests that cryo-thermal therapy offers a new therapeutic modality of remodeling the host’s immune environment to generate persistent anti-tumor memory for inhibition of metastasis.

## Results

### Cryo-thermal therapy induced strong long-lasting immune-mediated rejection of lung metastasis

Previously, we found that all treated primary tumors following cryo-thermal therapy decreased in size with necrotic scabs appearing in ~2 days. The tumors usually rubbed off in 1 week and the mice were in good health condition. Lung metastasis was markedly inhibited and long-term survival rates were achieved after cryo-thermal therapy^25^.

**Fig. 1:**
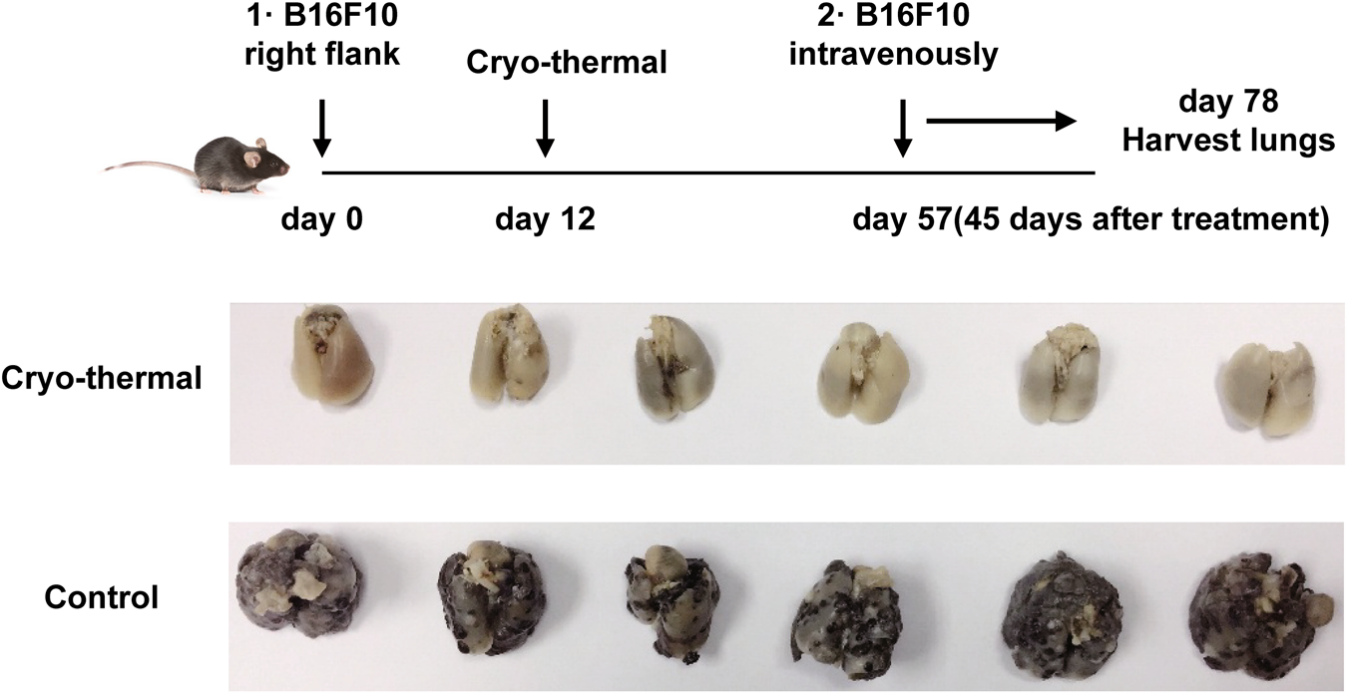
Cryo-thermal therapy protected mice from pulmonary metastatic B16F10 tumors. Photographic images of lungs from cryo-thermal-treated and B16F10 tumor-bearing mice, respectively. Upper: schematic of experimental design. Lower: photographic images of lungs

To verify whetherthe generated strong and long-lasting systemic immunologic memory response was a characteristic hallmark of the cryo-thermal therapy, the middle time point (45 days after treatment) was chosen. After 45 days, B16F10 melanoma cells were intravenously infused and lung nodules were enumerated on day 78 after tumor inoculation (Fig. 1). All control mice developed tumor nodules in the lungs. In contrast, lung tumor nodules were entirely controlled in long-term survivors treated by cryo-thermal therapy (Fig. 1). The results indicated that local cryo-thermal therapy generated strong immune memory responses that protected against tumor re-challenge and lung metastases.

### Cryo-thermal therapy induced activation and maturation of DCs

DCs are pivotal in initiating innate and adaptive immune responses. We hypothesized that mature DCs were induced following cryo-thermal therapy, which then initiated tumor*-*specific T-cell responses and long-term anti-tumor immunity.

**Fig. 2:**
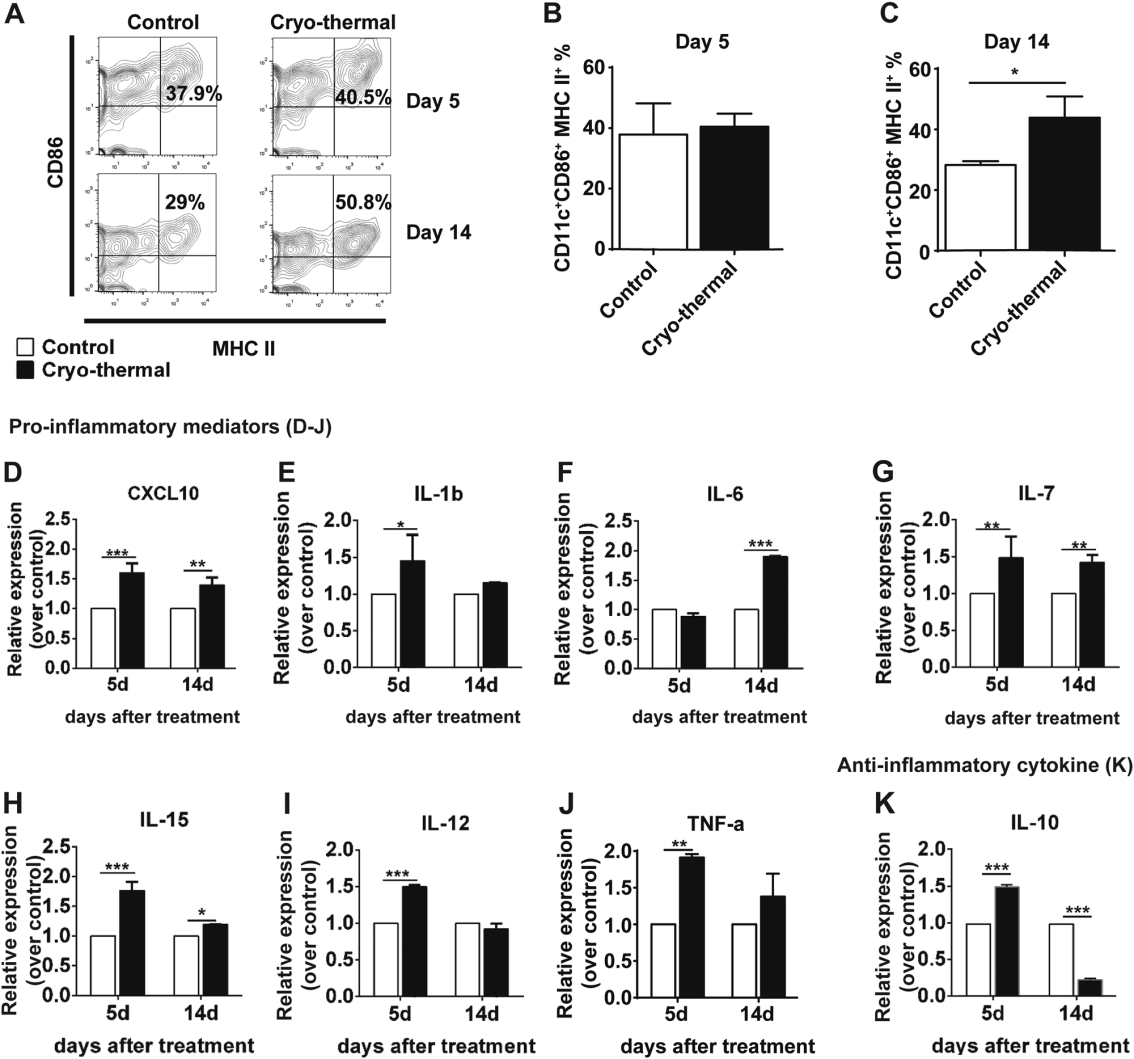
Cryo-thermal therapy induced phenotypically maturation of splenic DCs. The phenotype of immune cells collected from the spleen in the treated mice by cryo-thermal therapy and untreated tumor-bearing mice on day 5 and 14 were analyzed by flow cytometry. **a**–**c** Percentage of CD11c^+^CD86^+^MHC II^+^ DCs on day 5 and 14 were analyzed by flow cytometry; all data was shown as mean ± SD. *n* = 6 per group. Data for bar graphs were calculated using Student’s *t*-test. *p < 0.05. Total RNA was isolated from splenic CD11c^+^ DCs (purified from splenocytes using micro-bead kit) in cryo-thermal-treated mice on day 5 and 14. The mRNA in splenic CD11c^+^ DCs from tumor-bearing mice on day 17 and 26 after tumor inoculation was used as control. The expression level of *CXCL10* (**d**), *IL-1β* (**e**), *IL-6* (**f**), *IL-7*(**g**), *IL-15*(**h**), *IL-12p40*(**i**), *TNF-α* (**j**), and *IL-10* (**k**). Data were shown as mean ± SD. Data for bar graphs were calculated using two-way ANOVA. *p < 0.05; **p < 0.01; ***p < 0.001. *IL-12* refer to *IL-12p40* in the figure

The activation and maturation of DCs are required for both the upregulation of major histocompatibility complex (MHC) II antigen-presenting molecules and CD86 co-stimulatory molecules on the cell surface^26^. On day 14 after treatment, cryo-thermal therapy markedly induced high expression of CD86 and MHC II (on day 26 after tumor inoculation) (Fig. 2a–c).

Fully mature DCs are characterized by increased production of pro-inflammatory cytokines, which promotes anti-tumor T-cell activation and differentiation^16,27,28^. The level of the pro-inflammatory chemokine CXCL10 was significantly upregulated on day 5 and day 14. IL-12p40 mRNA expression was significantly upregulated on day 5. IL-7 and IL-15 (essential mediators that promote memory formation and maintenance^29,30^) mRNA was significantly upregulated on day 5 and maintained at a high level on day 14 (Fig. 2d–i). IL-1β were significantly upregulated in the cryo-thermal therapy group on day 5 but relatively decreased on day 14 (Fig. 2e). The IL-6 mRNA was not upregulated on day 5 but was significantly upregulated on day 14 (Fig. 2f). Tumor necrosis factor (TNF)-α was upregulated in the cryo-thermal therapy group on day 5 and stably expressed on day 14 (Fig. 2j). The cytokine IL-10 has a very important immuno-regulatory role in immune homeostasis^31^. The mRNA level of IL-10 was even lower on day 14 compared with that on day 5 (Fig. 2k). These findings show that cryo-thermal therapy triggers a distinct and stable pro-inflammatory phenotype in splenic DCs within 14 days following treatment.

As shown in Supplementary Fig. 2, the mRNA expression level of immune-inhibitory molecules (FOXO3, PD-L1, VEGFR2, and IDO1) in DCs were all significantly downregulated on day 14 following treatment. Altogether, these results suggest that cryo-thermal therapy persistently modulates the phenotypic and functional maturation of splenic DCs, while also increasing production of pro-inflammatory cytokines and downregulating the expression of immuno-inhibitory molecules from day 5 to 14 following cryo-thermal therapy.

### Cryo-thermal therapy re-educated immunosuppressive macrophages phenotypically

**Fig. 3:**
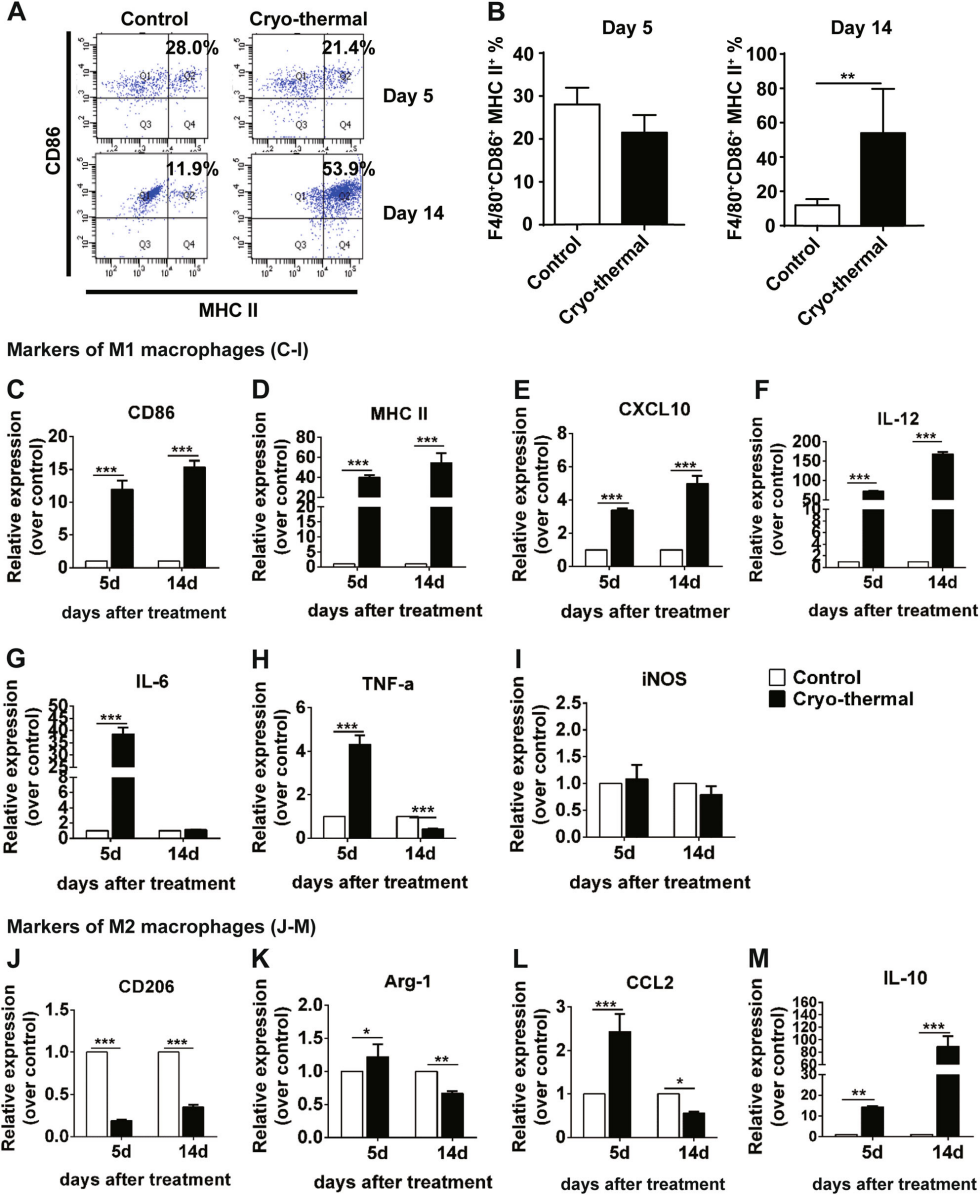
Cryo-thermal therapy re-educated immunosuppressive macrophages phenotypically. The phenotype of immune cells collected from the spleen in cryo-thermal-treated mice and untreated tumor-bearing mice on day 5 and 14 were analyzed by flow cytometry. **a**–**b** Percentage of CD11b^+^F4/80^+^CD86^+^MHC II^+^ macrophages on day 5 and 14 were analyzed by flow cytometry. All data was shown as mean ± SD. *n* = 6 per group. Data for bar graphs were calculated using Student’s *t*-test. **p < 0.01. Total RNA was isolated from splenic CD68^+^ macrophages (purified from splenocytes using micro-bead kit) in cryo-thermal-treated mice on day 5 and 14. The mRNA in splenic CD68^+^ macrophages from tumor-bearing mice on day 17 and 26 after tumor inoculation was used as control. The mRNA level of *CD86* (**c**), *MHC II* (**d**), *CXCL10* (**e**), *IL-12p40* (**F**), *IL-6* (**g**), *TNF-α* (**h**), *iNOS* (**i**), *CD206* (**j**), *Arg-1* (**k**), *CCL2* (**l**), and *IL-10* (**m**). Data were shown as mean ± SD. Data for bar graphs was calculated using two-way ANOVA. *p < 0.05; **p < 0.01; ***p < 0.001. *IL-12* refer to *IL-12p40* in the figure

Macrophages are also key regulators of initiating anti-tumor immune responses^9,32^. The phenotypic changes of macrophages were analyzed following treatment. Strikingly, M1 macrophage population in the spleen was increased on day 14 after cryo-thermal therapy, which was much higher than that in the control group (Fig. 3a, b).

M1 macrophages are characterized by high expression of MHC II, CD86, TNF-α, IL-6, IL-12, inducible nitric oxide synthase (iNOS), and the chemokine CXCL10. On day 5, cryo-thermal therapy elicited a marked increase in *CD86*, *MHC II*, *CXCL10*, *IL-12p40*, *IL-6*, and *TNF-α* mRNA levels (Fig. 3c–h). *CD86*, *MHC II*, *CXCL10*, and *IL-12p40* mRNA continued to increase on day 14 (Fig. 3c–f). Meanwhile, *IL-6* and *TNF-α* mRNA was drastically decreased from day 5 to 14 (Fig. 3g, h). No significant difference in the *iNOS* transcript level was found between the two groups (Fig. 3i).

The mRNA expression of the M2-associated transcripts was also determined. On day 5 after cryo-thermal therapy, *CD206* mRNA expression was markedly decreased (Fig. 3j). *Arg-1* mRNA expression was significantly increased (Fig. 3k). Chemokine *CCL2* and cytokine *IL-10* production were also markedly induced (Fig. 3l, m). On day 14, both *CD206* and *Arg-1* mRNA expression was lower than that in the control group (Fig. 3j, k). Interestingly, cryo-thermal therapy inhibited *CCL2* gene expression (Fig. 3l). *IL-10* mRNA expression level was also stably upregulated on day 14, but the level of *IL-10* was much lower than the level of *IL-12* (Fig. 3f–m). Together, these data demonstrated that cryo-thermal therapy triggered pro-inflammatory M1 macrophage polarization and suppressed M2 macrophages, offering in vivo evidence that cryo-thermal therapy can effectively stimulate the innate immune response.

We also evaluated the expression pattern of the molecules or receptors in splenic macrophages on day 14 after treatment. As shown in Supplementary Fig. 4, *VEGFR2*, *HO-1*, *IDO1*, *CCR2*, *CSF1R*, and *TRIALR* expression was greatly reduced as compared with the control group. The mRNA level of *PD-L1*, a marker for M1 macrophages^33^, was markedly upregulated as compared with the control group. Collectively, these results suggest that cryo-thermal therapy effectively triggers M1 macrophage polarization and suppresses M2 macrophages on day 14.

### Macrophage polarization toward the M1 phenotype remodeled the immune environment following cryo-thermal therapy

**Fig. 4:**
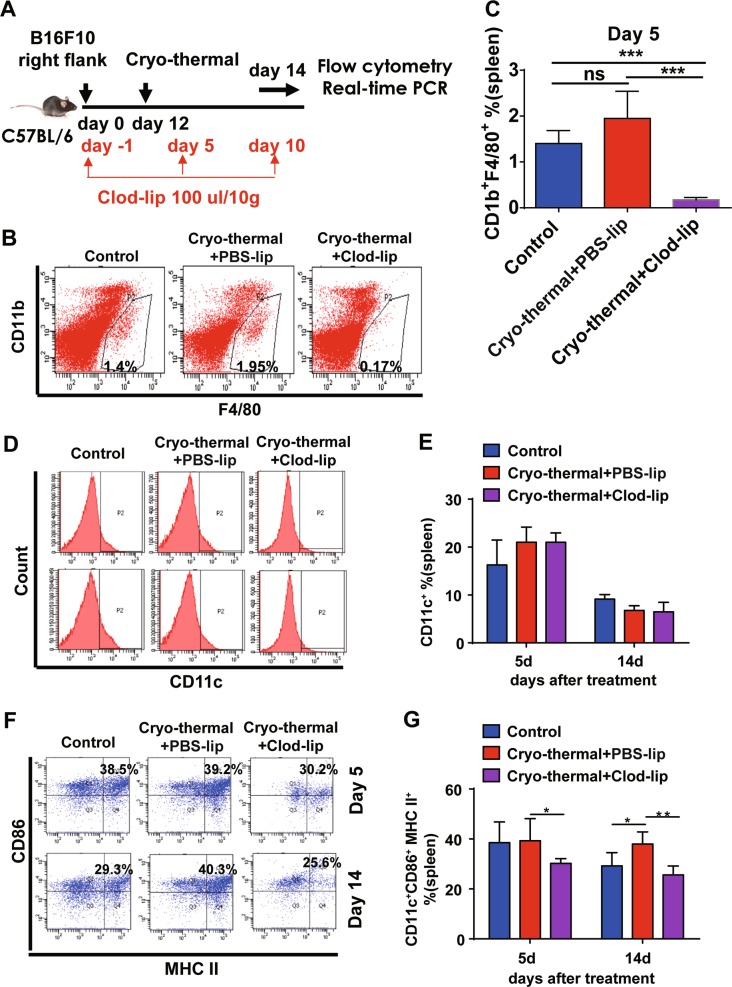
Cryo-thermal-re-educated macrophages remodeled host immune environment. **a** Schematic of experimental design. **b**, **c** The efficiency of macrophage depletion was assessed by flow cytometry assay. Data were shown as mean ± SD. *n* = 6 per group. ***p < 0.001. Data for bar graphs were calculated using one-way ANOVA. **d**–**g** The splenocytes were collected from the mice in cryo-thermal + PBS-lip, cryo-thermal + Clod-lip, and control groups, then the population of CD11c^+^ cells and percentage of CD86^+^MHC II^+^ DCs (gated on CD11c^+^ cells shown in **d**) on day 5 and 14 were analyzed by flow cytometry. All data were shown as mean ± SD. *n* = 6 per group. *p < 0.05 or **p < 0.01. Data for bar graphs were calculated using two-way ANOVA

As demonstrated above, cryo-thermal therapy triggered M1 macrophage polarization on day 5 (Fig. 3a–h), but the phenotypic and functional changes of DCs toward a mature state occurred on day 14 (Fig. 2), suggesting that cryo-thermal-induced M1 macrophage polarization occurred before cryo-thermal-induced DC maturation. In this regard, we hypothesized that DC maturation, which subsequently triggered a systemic anti-tumor effect, would be due to the re-education of macrophages to the M1 phenotype. To test this hypothesis, clodronate liposomes (Clod-lips) were used to deplete macrophages^34^ and the depletion of macrophages was confirmed in vivo on day 5 following treatment (Fig. 4a–c). We found that macrophage depletion resulted in less CD11c^+^ CD86^+^ MHC II^+^ matured DCs and downregulated the expression of pro-inflammatory cytokines in splenic DCs on day 5 and 14 (Fig. 4d–g, Supplementary Fig. 5). We next evaluated the changes of representative immuno-inhibitory or regulatory molecules in DCs by reverse transcriptase-PCR (RT-PCR) after macrophage depletion. On day 14, the mRNA levels of *STAT3* and *HO-1* in the cryo-thermal + Clod-lip group were significantly downregulated, whereas mRNA levels of *VEGFR2*, *IDO1*, and *FOXO3* were significantly upregulated (Supplementary Fig. 5D). These results suggest that activation and maturation of DCs induced by cryo-thermal therapy occur via a macrophage-dependent pathway.

In order to better understand the important role of cryo-thermal therapy-induced macrophage re-education to the M1 phenotype in long-term anti-tumor immune memory, the percentages of splenic CD4^+^ T cells, CD8^+^ T cells, and their markers were analyzed by flow cytometry, respectively.

**Fig. 5:**
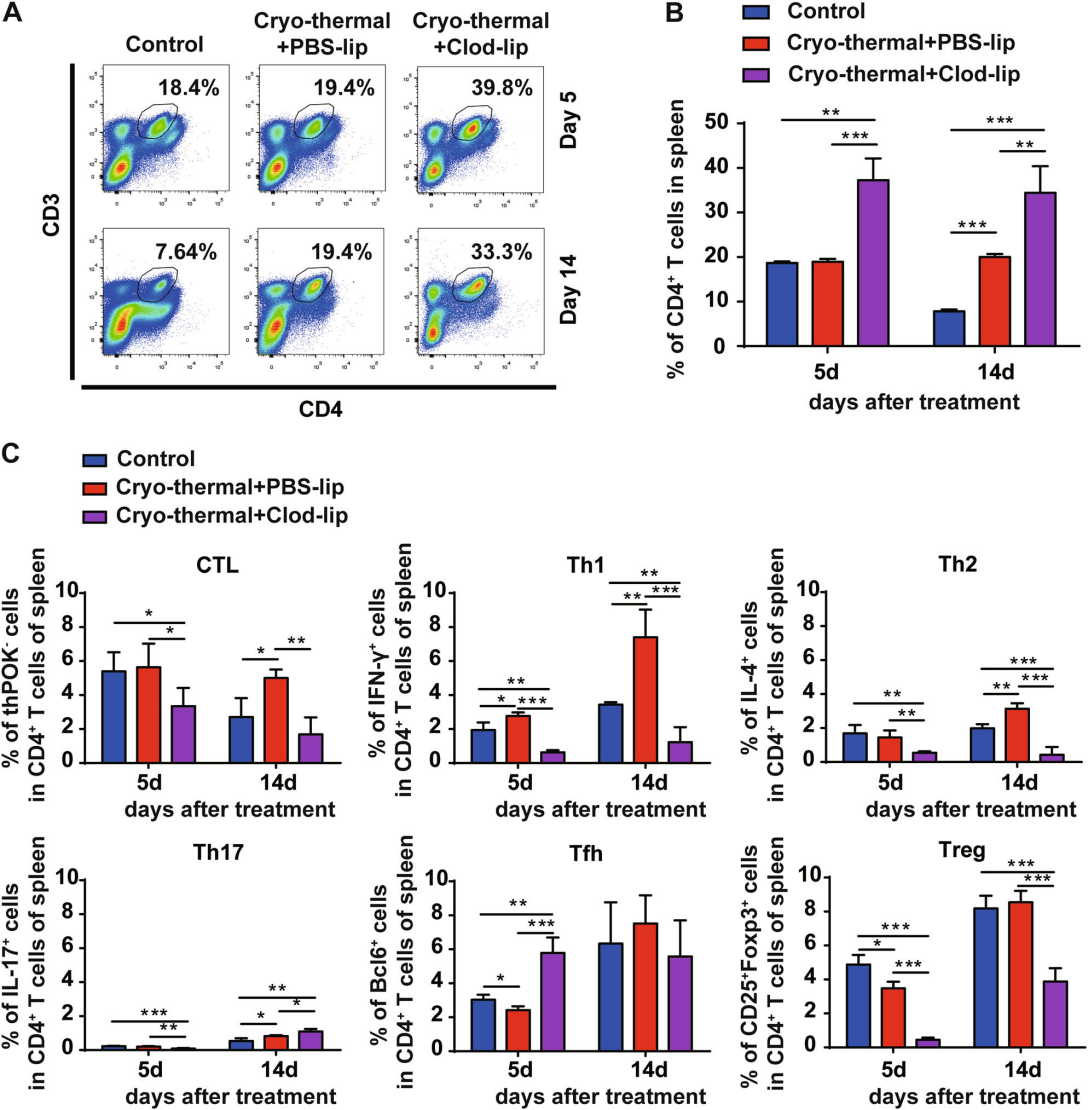
Cryo-thermal-re-educated macrophages remodeled host immune environment (continued). **a**, **b** The splenocytes were collected from the mice in cryo-thermal + PBS-lip, cryo-thermal + Clod-lip, and control groups, then the percentage of CD3^+^CD4^+^ T cells on day 5 and 14 were analyzed by flow cytometry. All data were shown as mean ± SD. *n* = 6 per group. **p < 0.01 or ***p < 0.001. Data for bar graphs were calculated using two-way ANOVA. **c** The level of thPOK (for CD4-CTL cells), IFN-γ (for Th1 cells), IL-4 (for Th2 cells), IL-17 (for Th17 cells), Bcl-6 (for Tfh cells), and FoxP3 (for Treg cells) in splenic CD4^+^ T cells were examined by flow cytometry. Data were shown as mean ± SD. Data for bar graphs were calculated using two-way ANOVA. *p < 0.05 or **p < 0.01, or ***p < 0.001

A significant increase in CD3^+^CD4^+^ T cells was found on day 5 and 14 following cryo-thermal therapy with Clod-lip treatment, respectively (Fig. 5a, b). On day 5, as compared with control, an increased proportion of Th1 cells and a decreased proportion of Tfh and Tregs were observed in the cryo-thermal + PBS-lip treatment group. However, when macrophages were depleted in cryo-thermal treated mice, the decreased levels of CD4-CTL, Th1, Th2, Th17, and Tregs were found, whereas the proportion of Tfh cells was increased as compared with cryo-thermal + PBS-lip treatment (Fig. 5c).

On day 14, the proportion of CD4-CTL, Th1, Th2, and Th17 cells were significantly increased after cryo-thermal + PBS-lip treatment as compared with control. However, the cryo-thermal + Clod-lip treatment completely abrogated the increased proportion of CD4-CTL, Th1, Th2, and Tregs (Fig. 5c). All of the above results indicate that macrophage depletion results in impaired CD4^+^ T-cell differentiation and induces CD4^+^ T-cell anergy, as confirmed by the decreased differentiation of CD4^+^ T cells into cytotoxic Th1 and CTLs.

**Fig. 6:**
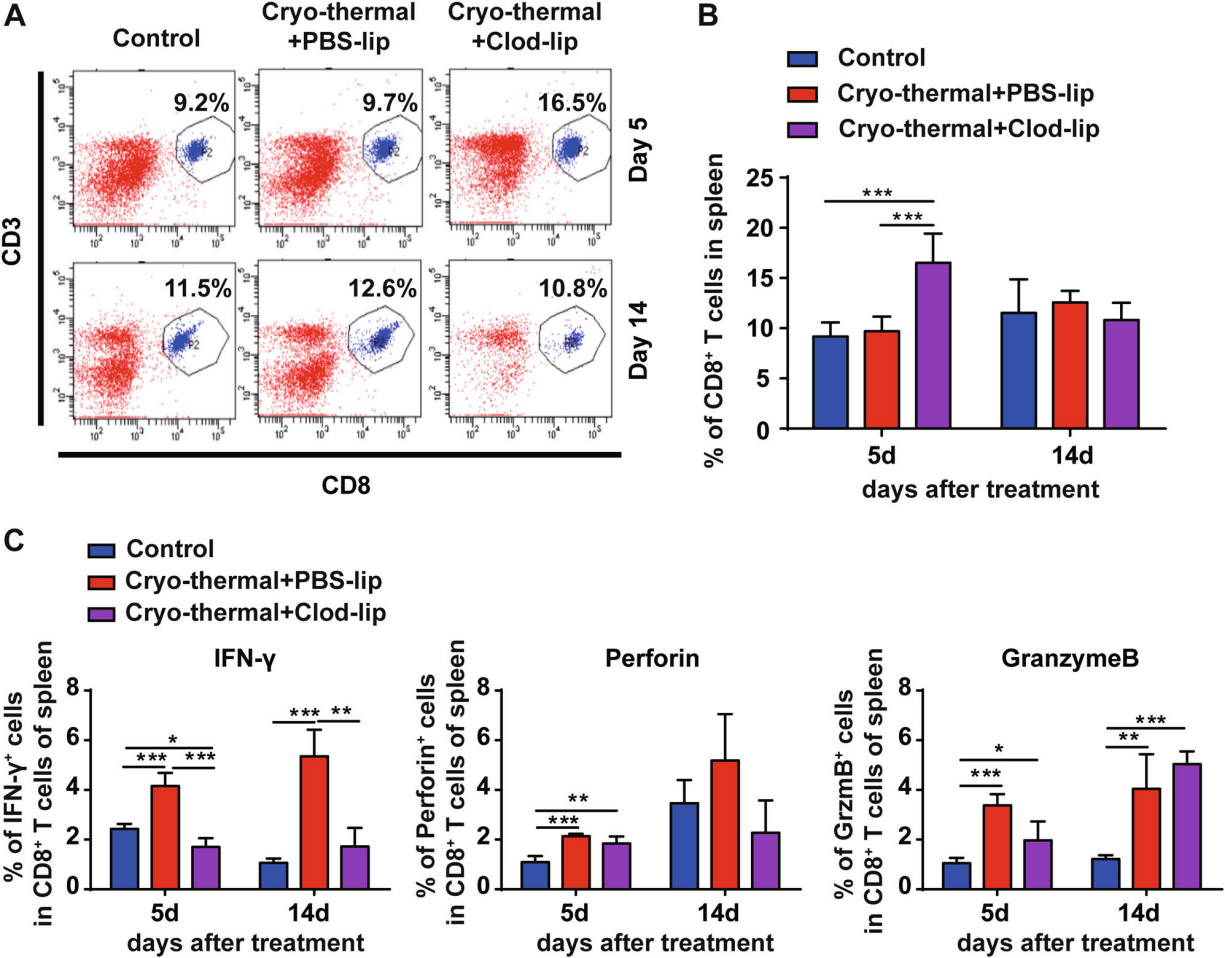
Cryo-thermal-re-educated macrophages remodeled host immune environment (continued). **a**, **b** The splenocytes were collected from the mice in cryo-thermal + PBS-lip, cryo-thermal + Clod-lip, and control groups, then the percentage of CD3^+^CD8^+^ T cells on day 5 and 14 were analyzed by flow cytometry. All data were shown as mean ± SD. *n* = 6 per group. ***p < 0.001, cryo-thermal + Clod-lip group compared with other groups. Data for bar graphs were calculated using two-way ANOVA. **c** The level of IFN-γ, perforin, granzyme-B in splenic CD8^+^ T cells were examined by flow cytometry. Data were shown as mean ± SD. Data for bar graphs were calculated using two-way ANOVA. *p < 0.05 or **p < 0.01, or ***p < 0.001

A significant increase in CD8^+^ T cells was also found on day 5 following cryo-thermal therapy with Clod-lip treatment (Fig. 6a, b). Macrophage depletion resulted in lower levels of IFN-γ and GzmB in CD8^+^ T cells on day 5, and much lower levels of IFN-γ in splenic CD8^+^ T cells on day 14 following cryo-thermal therapy (Fig. 6c). These results also indicated that macrophage depletion induced CD8^+^ T-cell anergy, as confirmed by the decreased cytotoxic effector function of CD8^+^ T cells. Together, these results suggest that cryo-thermal therapy can promote differentiation of CD4^+^ T cells into cytotoxic Th1, CTL cells, and differentiation of CD8^+^ T cells into cytotoxic effector T cells via macrophage re-education toward the M1 phenotype.

### Cryo-thermal-re-educated M1-polarized macrophages restored the phenotypic and functional maturation of tumor-bearing DCs in vitro

We surmised whether cryo-thermal-induced macrophage polarization to the M1 phenotype would effectively reshape immature DCs to fully mature DCs in vitro. As expected, the tumor-bearing DCs co-cultured with cryo-thermal macrophages were highly matured, as demonstrated by the increased percentage of CD11c^+^CD86^+^MHC II^+^ DCs along with the higher relative expression of *IL-6*, *TNF-α*, *CXCL10*, *IL-1β*, *IL-12p40*, and *IL-15* mRNA (Supplementary Fig. 6A-B). Our data revealed that cryo-thermal-induced repolarization of macrophages to the M1 phenotype could promote phenotypic and functional maturation of DCs in vitro.

### Cryo-thermal-induced repolarization of macrophages to the M1 phenotype was required for promoting polyfunctional CD4^+^ and CD8^+^ T cells with cytotoxic effector function in vitro

We posited that the reshaped DCs would promote the functional plasticity of tumor-bearing T cells. Therefore, in vitro experiments were performed to investigate the potential relationships among DCs, macrophages, and T cells.

As expected, mature DCs induced by cryo-thermal therapy directly promoted the proliferation of CD4^+^ T cells. Both tumor-bearing macrophages and cryo-thermal macrophages could directly inhibit CD4^+^ T-cell proliferation. Moreover, the effect of tumor-bearing macrophages on inhibiting proliferation of CD4^+^ T cells was much stronger than that of cryo-thermal-induced M1 macrophages (Supplementary Fig. 7A–B).

Furthermore, the phenotypically matured splenic DCs following cryo-thermal therapy could moderately promote tumor-bearing CD4^+^ T-cell differentiation into Th1, CD4-CTL, and Th2 sub-lineages. Moreover, cryo-thermal-induced macrophage polarization to the M1 phenotype was required for phenotypically matured splenic DCs to promote tumor-bearing CD4^+^ T-cell differentiation into Th1 and CD4-CTL. Unexpectedly, tumor-bearing macrophages and cryo-thermal-induced M1 macrophage polarization could promote CD4^+^ T-cell differentiation into CD4-CTL and Th17, but the effect of cryo-thermal-induced M1 macrophage was much stronger than that of tumor-bearing macrophages (Supplementary Fig. 7C). In summary, our data revealed that cryo-thermal-induced repolarization of macrophages to the M1 phenotype could not only directly promote CD4^+^ T-cell differentiation into CTLs but could also trigger DC maturation to enhance CD4^+^ T-cell differentiation into Th1 and CD4-CTLs

Our results also indicate that cryo-thermal-re-educated macrophages can promote the proliferation of tumor-bearing CD8^+^ T cells with a cytotoxic functional phenotype (Supplementary Fig. 8A–C).

### Melanoma-specific CD4-CTLs was induced by cryo-thermal-re-educated M1 macrophages

We next examined the cytotoxicity of splenic CD4^+^ T cells and CD8^+^ T cells against tumor cells in vitro by a CCK-8 assay. Interestingly, the cell viability of B16F10 cells was significantly lower than that in tumor-bearing CD4^+^ T cells when cryo-thermal-CD4^+^ T cells were cultured with B16F10 cells at ratios of 1:1 and 2:1 (Supplementary Fig. 9A). Meanwhile, there was no significant difference in the cell viability among cryo-thermal-CD8^+^ T cells, cryo-thermal-Clod-lip-CD8^+^ T cells, and tumor-bearing CD8^+^ T cells group (Supplementary Fig. 9C). The results confirmed those from our previous study that durable anti-tumor immunity was mainly dependent on CD4^+^T cells^27^. However, the cytolysis of CD4^+^ T cells was abrogated when macrophages were depleted by Clod-lip (Supplementary Fig. 9A). These results indicate that cytolysis of CD4^+^ T cells induced by cryo-thermal therapy is mediated by cryo-thermal-re-educated M1 macrophages. Furthermore, the splenic CD4^+^ and CD8^+^ T cells from the three groups all could not inhibit mouse 4T1 breast cancer cells and even promoted the proliferation of 4T1 cells (Supplementary Fig. 9B and 9D). These data suggest that the cryo-thermal-induced polarization of macrophages to the M1 phenotype has a critical role in melanoma-specific cytolysis of CD4^+^ T cells.

### Cryo-thermal-induced macrophage polarization to the M1 phenotype was crucial for triggering anti-tumor immune memory

**Fig. 7:**
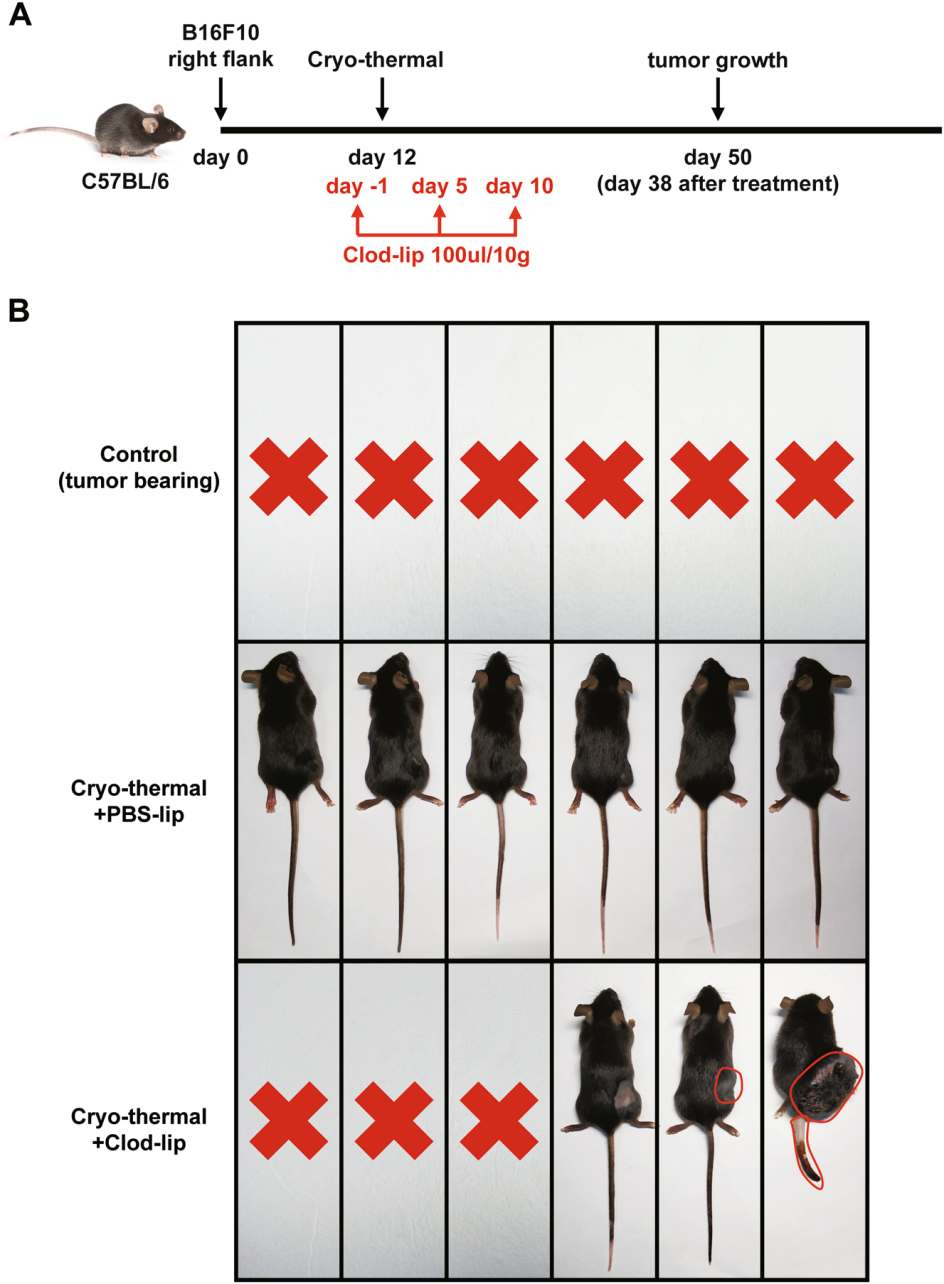
Cryo-thermal-induced macrophage polarization to the M1 phenotype was crucial for triggering anti-tumor memory immunity. **a** Schematic of experimental design. Macrophage depletion was achieved by intraperitoneal injection of Clod-lip (red arrows indicated Clod-lip injection) on day 1 before cryo-thermal therapy and on day 5, 10 after cryo-thermal therapy, respectively. The tumor growth was tracked on day 50 after tumor inoculation (38 days after the cryo-thermal therapy). **b** Photographic images of tumor growth in cryo-thermal + PBS-lip, cryo-thermal + Clod-lip, and control groups

To demonstrate the importance of macrophage polarization to the M1 phenotype in the induction of strong and long-lasting systemic anti-tumor immunity mounted by cryo-thermal therapy, Clod-lips were used to deplete macrophages in cryo-thermal-treated mice (Fig. 7a). As shown in Fig. 7b, all tumor-bearing mice were dead on day 50 following the tumor inoculation, whereas all of the cryo-thermal-treated mice injected with phosphate-buffered saline (PBS) liposomes did not develop tumors 38 days after the treatment. Macrophage depletion resulted in the death of three mice due to recurrence in situ and lung metastasis. Recurrence in situ was observed in two other mice and tumor metastasis on the tail was observed in one mouse among the two on day 38 after cryo-thermal therapy. These data suggest that macrophage polarization to the M1 phenotype has an important role in cryo-thermal induction of strong and long-lasting anti-tumor immune memory.

## Discussion

**Fig. 8:**
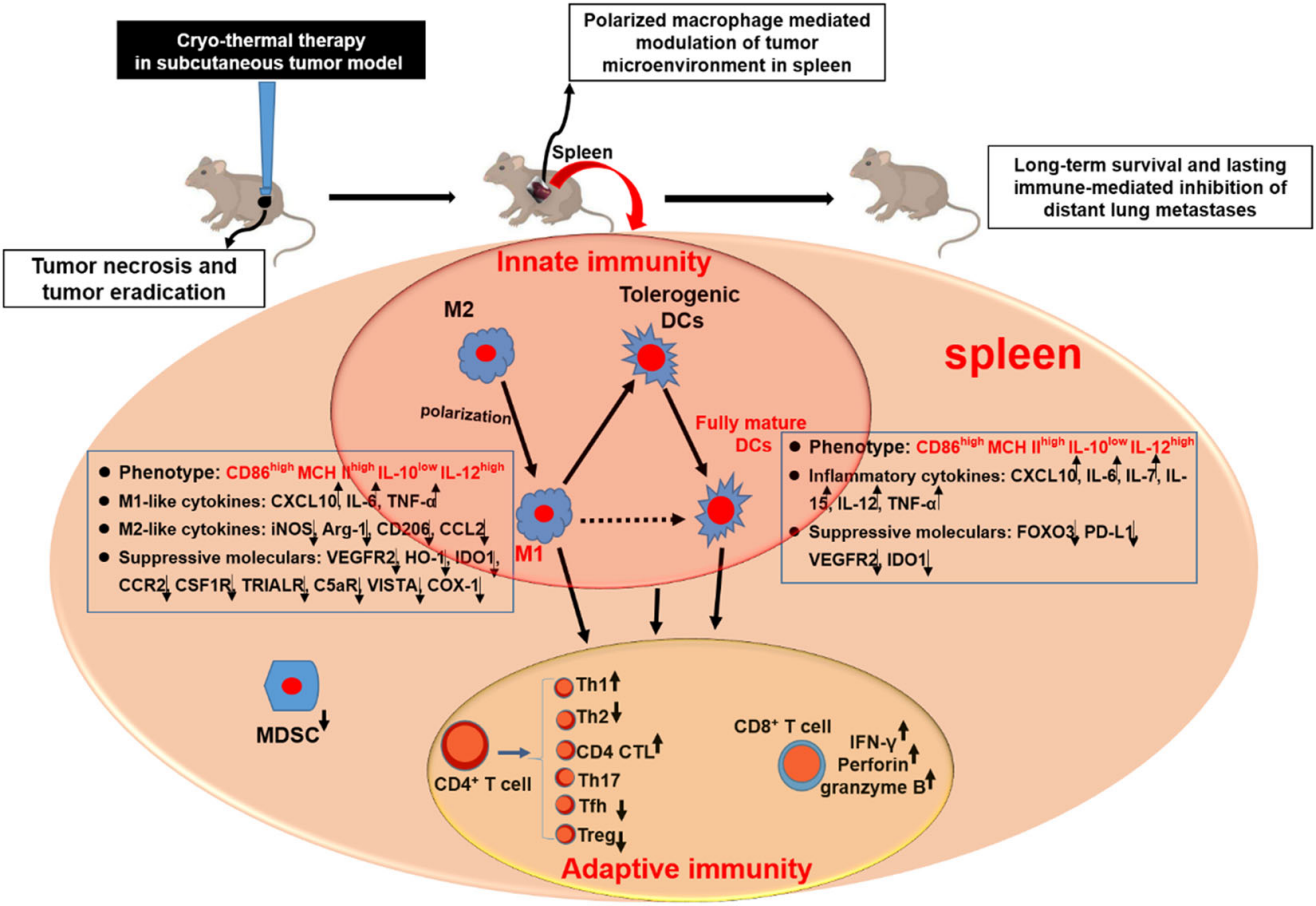
Schematic representation of cryo-thermal therapy drove macrophages polarization toward M1 phenotype that remodeled host immune environment triggering the durable anti-tumor memory immunity. Cryo-thermal therapy triggered the macrophage polarization towards M1 phenotype. Cryo-thermal therapy also induced the phenotype and functional maturation of DCs. Moreover, cryo-thermal-induced M1 polarization macrophage could trigger DC maturation, CD4^+^ T cell differentiation into Th1 and CTL sublineages, and the generation of cytotoxic CD8^+^ T cells, which lead to long-lasting adaptive immune-mediated inhibition of distant lung metastases

In this study, we revealed that cryo-thermal therapy remodeled the immune environment, triggering durable anti-tumor immune memory to inhibit metastasis in a B16F10 melanoma model. Cryo-thermal therapy persistently modulated the phenotypic and functional maturation of DCs and re-educated macrophage polarization to the M1 phenotype. Moreover, our studies demonstrated that cryo-thermal-induced macrophage polarization to the M1 phenotype was exclusively responsible for the subsequent DC activation and maturation, CD4^+^ T-cell differentiation into Th1 and CTL sub-lineages, and the generation of cytotoxic CD8^+^ T cells. Our findings further emphasize that cryo-thermal-induced macrophage polarization to the M1 phenotype is essential to mediate anti-tumor immune memory and lead to long-term survival (Fig. 8).

The generation of a spontaneous anti-tumor T-cell response depends upon innate immune activation^35^. Innate immune cells, such as M1 macrophages and matured DCs, are capable of bridging innate and adaptive immune responses against tumors^9^. Cryo-thermal therapy persistently elicited fully mature DCs on day 14 after the treatment. The splenic mature DCs in cryo-thermal-treated mice expressed high levels of pro-inflammatory cytokines and chemokines, and the phenotypic and functional changes of splenic DCs from an immature to a fully mature state from day 5 to 14 following cryo-thermal therapy would modulate the host immune environment and finally shape systemic anti-tumor immune memory. On the other hand, cryo-thermal therapy triggered M1 macrophage polarization in treated mice on day 5 and efficiently induced M1 macrophage differentiation on day 14. Long-term memory anti-tumor immunity can be generated when the right cells and soluble cytokines are present^36^. When the innate immune cells exhibiting proper immune-stimulatory function were presented, the re-educated macrophages and reshaped DCs, as important initiators, determined the CD4^+^ T-cell differentiation and CD8^+^ T-cell cytotoxicity^27^, and a robust T-cell-mediated long-lasting immune response was generated.

However, the immunogenic role of mature DCs and M1 macrophages was not isolated; there was cross-talk between macrophages and DCs, which would be a critical immunologic process in regulating the whole immune defense against tumors following cryo-thermal therapy. Although studies have shown that DCs are professional APCs and can trigger T-cell activation and differentiation^12,37^, our findings indicated that cryo-thermal-induced repolarization of macrophages to the M1 phenotype had a regulatory role in stimulating DC activation and maturation. Macrophage depletion downregulated the expression of co-stimulatory molecules and the production of inflammatory cytokines in DCs, all of which are critical for T-cell activation, differentiation, and expansion^27^. Moreover, splenic macrophages isolated from cryo-thermal-treated mice effectively reshaped DCs derived from tumor-bearing mice to mature DCs. Furthermore, M1 macrophages promote Th1 immune responses, improve T-cell recruitment, and suppress the activities of M2 macrophages ^38,39^. In this study, cryo-thermal therapy-mediated polarization of macrophages into the M1 phenotype could directly promote anti-tumor CD4^+^ and cytotoxic CD8^+^ T-cell activation. These findings identified that cryo-thermal therapy stimulated macrophage DC cross-talk to activate anti-tumor CD4^+^ and cytotoxic CD8^+^ T cells.

Targeting macrophage phenotype transformation has been regarded as a novel strategy for cancer treatment^40^. In this study, cryo-thermal therapy induced macrophage polarization into the M1 phenotype in the treated mice, as demonstrated by the potent expression of acute inflammatory cytokines and downregulating M2-like associated markers in macrophages. For example, the pivotal feature of IL-6 induced by cryo-thermal therapy was high and transient in expression. An IL-6-rich acute inflammatory response was required to endow DC phenotypic maturation leading to the functional differentiation of CD4^+^ T cells^24^. In this study, the increased expression of IL-6 in macrophages appeared on day 5 following cryo-thermal therapy, whereas the increased expression of IL-6 in DCs was found on day 14. Therefore, we proposed that a cascade of immunological events was initiated by cryo-thermal therapy and macrophages responded much more quickly than DCs. More importantly, cryo-thermal therapy greatly increased the expression level of the chemokine CXCL10 in macrophages, which attracts activated T cells and natural killer cells^41^. IL-10 is a negative regulator of IL-12-mediated immunological activities and has very important immuno-regulatory roles in host immune homeostasis^31^. In this study, cryo-thermal therapy was able to drive macrophage polarization to the M1 phenotype, which was represented by high IL-12p40 and low IL-10 levels. On day 5 following cryo-thermal therapy, the elevated expression of CCL2 attracts monocytes^42,43^, and monocytes then differentiate into full mature DCs or M1-like macrophages in the ideal immunogenic and immunostimulatory environment. Altogether, our studies showed that M1 macrophage polarization was triggered and M2 macrophage polarization were delayed after cryo-thermal therapy, thus facilitating anti-tumor immune environment undefined. As macrophages have a very important role as initiators of cryo-thermal-mediated anti-tumor immune responses, the mechanism underlying cryo-thermal-mediated M1 polarization of macrophages needs to be further studied.

In summary, cryo-thermal therapy could drive M1 macrophage polarization to remodel the host immune environment by promoting subsequent DC activation and maturation, CD4^+^ T-cell differentiation into Th1 and CTL, and generation of cytotoxic CD8^+^ T cells, leading to effective anti-tumor immune memory capable of inhibiting distant tumor metastasis. Thus, cryo-thermal therapy may represent a new strategy to promote macrophage phenotype transformation for cancer treatment.

## Materials and methods

### Animal model

Female C57BL/6 mice were obtained from Shanghai Slaccas Experimental Animal Co., Ltd (China) and used for experimental study at the age of 6–8 weeks. They were housed in isolated cages and a 12 h light/dark cycle environment, feeding with sterile food and acidified water with pH value kept at 2.5–2.8. All animal experiments were approved by the Animal Welfare Committee of Shanghai Jiao Tong University and experimental methods were performed in accordance with the guidelines of Shanghai Jiao Tong University Animal Care (approved by Shanghai Jiao Tong University Scientific Ethics Committee). Murine B16F10 cells (donated by Professor Weihai Yin at Med-X Research Institute, Shanghai Jiao Tong University) were cultured in RPMI 1640 medium (Hyclone, USA) supplemented with 10% fetal bovine serum (FBS), plus 100 U/mL penicillin, and 100 g/ml streptomycin (Shanghai Sangon, China). To prepare the tumor-bearing mice, ~5 × 10^5^ cells were injected subcutaneously into the right femoral region of each mouse. Tumor sizes were measured every 2–3 days and its volume was estimated using the following formula: *V* (cm^3^) = *π* × *L* (major axis) × *W* (minor axis) × *H* (vertical axis)/6.

### The thermal therapy procedures

The system developed in our laboratory was composed of liquid nitrogen for cooling and radiofrequency (RF) for heating^44^. To reduce the effect of contact thermal resistance and obtain a constant thermal delivery during the treatment, a probe was designed with a cylinder-shaped tip of 10 mm in diameter for the thermal therapy of subcutaneous tumor^45^. Subcutaneous injection of B16F10 melanoma cells into C57BL/6 mice leads to form primary tumors in 7–9 days and spontaneous metastasis in the lungs^46^. Twelve days after tumor inoculation, when the tumor volume reached about 0.2 cm^3^, mice were divided into two groups: tumor-bearing group without the treatment (control) and cryo-thermal group with freezing at the temperature of − 20 °C for 5 min followed by RF heating at the temperature of 50 °C (the simulated temperature distribution during the RF heating process was performed previously^24^) for 10 min on primary tumor (cryo-thermal). The mice were anesthetized with intraperitoneal (i.p.) injection of 1.6% pentobarbital sodium (0.5 ml/100 g, Sigma-Aldrich). The tumor site was sanitized with alcohol and iodine tincture before the treatment. All of the procedures were performed aseptically.

### Tumor re-challenge analysis

To investigate the distal effect of the treatment, primary B16F10 melanoma tumor in C57BL/6 mice were treated and 45 days later the treated mice were intravenously infused with 1 × 10^5^ B16F10 melanoma cells, and lung tumor nodules were enumerated (*n* = 6 per group). Tumor growth was measured every day using calipers and tumor volumes were estimated.

### Preparation of a single-cell suspension and FACS analysis

Mice were killed on day 5 and 14 after cryo-thermal therapy and the spleen was collected (*n* = 4 per group at each time point). Single-cell suspension of splenocytes was prepared using GentleMACS™ dissociator (Miltenyi Biotec) and then treated with erythrocyte-lysing reagent containing 0.15 M NH_4_Cl, 1.0 M KHCO_3_, and 0.1 mM Na_2_EDTA to remove red blood cells. Staining antibodies including CD11b-FITC (clone M1/70), Gr-1-PE (clone RB6-8C5), F4/80-APC (clone BM8), CD86-PE (clone GL-1), IA-IE-percp-cy5.5 (clone M5/114.15.2), CD11c-FITC (clone N418), CD3-FITC (clone 145-2C11), CD4-APC/Cy7 (clone RM4.5), CD8-APC/Cy7 (clone 53-6.7), IFN-γ-BV510 (clone XMG1.2), IL-4-BV421 (clone 11B11), IL-17-PE (clone TC11-18H10.1), Perforin-PE (clone S16001B), Granzyme-B-AF647 (clone GB11), Bcl-6-percp-cy5.5 (clone 7D1), CD25-PE/Cy7 (clone 3C7), and Foxp3-PE (clone MF-14) were purchased from Biolegend (USA). ThPok-AF647 (clone T43-94) was purchased from BD Bioscience (USA). For cell surface staining, splenocytes were stained with antibodies as described above, for 30 min at 4 °C. For intracellular staining, splenocytes were stimulated for 4 h with Cell Activation Cocktail (phorbol-12-myristate 13-acetate, ionomycin, and Brefeldin A) (Biolegend) according to the manufacturer’s protocol. Cells were incubated with anti-FcγR antibody, followed by surface staining with antibodies binding cell-specific surface marker, then fixed and permeabilized according to the manufacturer’s instructions and incubated with antibodies binding specific intercellular marker. For transcription factor staining, splenocytes were incubated with anti-FcγR antibody to block unspecific binding before adding antibodies. Then cell surface staining and transcription factor staining was carried out according to the manufacturer’s protocol using the True-Nuclear™ Transcription Factor Buffer Set. True-Nuclear™ Transcription Factor Buffer Set, Fixation Buffer, Intracellular Staining Permeabilization Wash Buffer, and Cell Activation Cocktail (with Brefeldin A) were all purchased from Biolegend. The cells were analyzed on a FACS Aria II cytometer (BD Biosciences) and the data were analyzed using FlowJo software.

### Isolation of CD68^+^macrophages, CD11c^+^ DCs, CD4^+^, and CD8^+^ T cells

For isolation of T cells, spleens from the treated and tumor-bearing C57BL/6 mice were collected on day 5 and 14 after the treatment (*n* = 4 mice per group at each time point), respectively, and splenocytes were prepared using GentleMACS™ dissociator (Miltenyi Biotec) and passed through a 40 μm nylon filter. CD4^+^ and CD8^+^ T cells were purified from splenocytes using Easysep mouse CD4^+^ and CD8^+^ T cell Enrichment Kits (StemCell Technologies, Vancouver, BC, Canada) according to the manufacturer’s instructions. CD4^+^ and CD8^+^ T cells with a purity of 90% were used for experiments. DCs were isolated by DC isolation micro-bead kit (Easysep^TM^ CD11c positive selection kit, StemCell Technologies, Vancouver, BC, Canada) according to the manufacturer’s instructions. For CD68^+^ macrophage isolation, splenocytes were plated in Dulbecco’s modified Eagle’s medium supplemented with 10% FBS at 37 °C in a humidified 5% CO_2_ incubator for 1 h, then the supernatant fraction was poured off and the adherent splenocyte fraction was processed for CD68^+^ macrophage isolation by using EasySep^TM^ PE positive selection kit (StemCell Technologies, Vancouver, BC, Canada) according to the manufacturer’s instructions.

### RNA isolation and real-time PCR

Total RNA was prepared from purified CD68^+^ macrophages and CD11c^+^ DCs using TRIzol Reagent (TaKaRa, Dalian, China). Absorbance at 260/280 nm for mRNA purity at a ratio above 1.9 was achieved for all samples used. cDNA was made using a PrimerScript RT reagent kit with gDNA Eraser (TaKaRa, Dalian, China). Quantitative real-time PCR (RT-PCR) was performed on ABI 7900HT sequence detection system and SDS software (Applied Biosystems, Foster City, CA, USA) using SYBR Premix Ex Taq (TaKaRa) and samples were amplified in 384-well plates. The mRNA in splenic CD68^+^ macrophages and CD11c^+^ DCs from tumor-bearing mice were used as the control, to compare the marker profiles of splenic CD68^+^ macrophages and CD11c^+^ DCs from the treated mice, expressed as a fold difference, respectively. The primer sequences of mouse genes are presented in Supplementary Table 1. Relative expression levels of mRNA for each gene were normalized to glyceraldehyde 3-phosphate determined by using the Ct value and assessed using relative quantification (ΔΔCt method). All experiments were performed in triplicates.

### Macrophage depletion

Macrophage depletion was performed by i.p. injection of a loading dose of 0.1 ml/10 g of the clodronate-encapsulated lipsome (http://www.ClodronateLiposomes.com) every 5 days from day 1 before treatment to day 14 after cryo-thermal therapy. Control liposomes contained PBS. The efficiency of macrophage depletion was assessed by flow cytometry assay. In another experiment, macrophage depletion was performed with clodronate-encapsulated lipsome injection from day 1 before treatment to day 14 after cryo-thermal therapy and the survival of cryo-thermal-treated mice was observed (*n* = 6 per group).

### In vitro cell co-culture

For understanding the role of cryo-thermal-induced re-educated macrophages in DCs phenotypic maturation and activation, the isolated CD68^+^ macrophages from tumor-bearing group (termed as tumor-bearing macrophage) and cryo-thermal group (termed as Cryo-thermal macrophage) were co-cultured with CD11c^+^ DCs from the tumor-bearing mice (termed as tumor-bearing DCs) at a ratio of 1:1 for 24 h; the co-cultured DC cells were detected by flow cytometry and purified for quantitative RT-PCR assay. The tumor-bearing DCs were taken as the control group.

The in vitro experiments were performed for understanding the potential relationships among DCs, macrophages, and T cells. The splenic CD11c^+^ DCs were isolated from tumor-bearing mice (termed as tumor-bearing DCs) on day 26 following the tumor inoculation and cryo-thermal group on day 14 following the treatment (termed as Cryo-thermal-DCs), respectively. The CD4^+^ T cells and CD8^+^ T cells from the spleen of tumor-bearing mice (termed as tumor-bearing CD4^+^ T cells and tumor-bearing CD8^+^ T cell) were also isolated on day 26 following the tumor inoculation. The tumor-bearing CD4^+^ T cells and CD8^+^ T cells were taken as the control (termed as group A). The splenic tumor-bearing DCs were co-cultured with tumor-bearing CD4^+^ T cells and tumor-bearing CD8^+^ T cells at a ratio of 1:5 in 96-well plate for 24 h. The splenic CD11c^+^ DCs from cryo-thermal-treated mice were co-cultured with CD4^+^ T cells and CD8^+^ T cells from the spleen of tumor-bearing mice at a ratio of 1:5 in 96-well plate for 24 h, respectively. The splenic CD11c^+^ DCs were isolated from cryo-thermal-treated mice received Clod-lip as described above (termed as Clod-lip-cryo-thermal-DCs). Then, Clod-lip-cryo-thermal-DCs were co-cultured with tumor-bearing CD4^+^ T cells and tumor-bearing CD8^+^ T cells. In order to understand the role of cryo-thermal-induced re-educated macrophages in interacting with both CD4^+^T and CD8^+^T cells directly, the splenic CD68^+^ macrophages from tumor-bearing mice or cryo-thermal-treated mice were also co-cultured with CD4^+^ T cells and CD8^+^ T cells from the spleen of tumor-bearing mice as described above, respectively. The co-cultured CD4^+^ T cells and CD8^+^ T cells were detected by flow cytometry.

### Proliferation assay

Carboxyfluorescein succinimidyl ester (CFSE) was used to measure the cell proliferation activity of tumor-bearing CD4^+^ and CD8^+^ T cells after being co-cultured with DCs or macrophages. Briefly, 1 × 10^7^ CD4^+^ or CD8^+^ T cells were incubated with CFSE at a final concentration of 5 μM/ml for 30 min and washed twice, then co-cultured with DCs or macrophage for 24 h. CFSE dilution was measured by flow cytometry. As the CFSE signal is diluted with each cell division, cells exhibiting a high CFSE fluorescence intensity is considered not to have proliferation and a low CFSE fluorescence intensity is considered to have proliferation.

### Cytotoxic assay

Cytotoxicity of splenic CD4^+^T cells and CD8^+^T cells against mouse B16F10 melanoma cells and mouse mammary carcinoma 4T1 cells were evaluated using a colorimetric cell-counting kit (CCK-8, Dojindo Laboratories), according to the manufacturer’s instructions. Briefly, splenic CD4^+^T cells from cryo-thermal-CD4^+^ T-cell group, cryo-thermal-Clod-lip-CD4^+^ T-cell group, tumor-bearing CD4^+^ T-cell group or CD8^+^ T cells from cryo-thermal-CD8^+^ T-cell group, cryo-thermal-Clod-lip-CD8^+^ T-cell group, and tumor-bearing CD8^+^ T-cell group were co-cultured in triplicate with B16F10 cells or 4T1 cells (2 × 10^4^ cells/well) at the ratio of 1:1, 2:1, 4:1, and 8:1 for 24 h at 37 °C in 5% CO_2_, respectively. Then, the supernatants were poured off to discard T cells and exposed to cell culture media with CCK-8 solution (10%) for 2 h at 37 °C. Furthermore, the absorbance at 450 nm was measured on a microplate reader. The B16F10 cells and 4T1 cells were used as tumor controls and the CCK-8 solution with cell culture media alone was used as blank control, respectively. The percentage of cell viability of tumor cells was calculated as [(experimental − blank control)]/[(tumor control − blank control)] × 100%.

### Statistical analysis

The Student’s *t*-test and one-way analysis of varianec (ANOVA) were used for statistical comparisons using Graph Pad Prism 6. Figures denoted statistical significance of *p < 0.05, **p < 0.01, and ***p < 0.001, or ^&^*p* < 0.05, ^&&^*p* < 0.01, or ^&&&^*p* < 0.001. Flank tumor growth curves were analyzed using two-way ANOVA. To assess survival differences, Kaplan–Meier curves were produced and analyzed by log-rank tests. Results were expressed as mean ± SD.

## Supplementary information

Supplemental table 1. Primer sequences of various genes in this study

Supplemental Figure 1. The change of splenic CD11c^+^ DCs

Supplemental Figure  2. The expression of immunoinhibitory or regulatory molecules on splenic CD11c^+^ DCs after cryo-thermal therapy

Supplemental Figure 3. The change of splenic CD11b^+^F4/80^+^ macrophages

Supplemental Figure 4. The expression of immunoinhibitory or regulatory molecules on splenic CD68^+^ macrophages after cryo-thermal therapy

Supplemental Figure 5. The expression of pro-inflammatory cytokines, immunoinhibitory or regulatory molecules on splenic CD11c^+^ DCs after cryo-thermal therapy plus Clod-lip treatment

Supplemental Figure 6. Cryo-thermal-re-educated splenic macrophages restored the phenotypic maturation of tumor-bearing DCs in vitro

Supplemental Figure 7. Cryo-thermal-re-educated splenic macrophages were required for promotion of functional polarized CD4^+^ T cells in vitro

Supplemental Figure 8. Cryo-thermal-induced macrophage polarization to the M1 phenotype was required for promoting cytotoxic CD8^+^ T cells in vitro

Supplemental Figure 9. Effect of specific cytotoxic T cells (CTLs) mediated by CD4^+^ T cells after cryo-thermal therapy

Supplemental figure legends
