# Supplementary material for: Cryo-thermal therapy induces macrophage polarization for durable anti-tumor immunity
Source: Cell Death Dis. 2019 Mar 4;10(3):216. doi: 10.1038/s41419-019-1459-7 (PMC6399266; doi:10.1038/s41419-019-1459-7)
Supplement: Supplementary file 1 — Supplemental table 1. Primer sequences of various genes in this study [file 41419_2019_1459_MOESM1_ESM.docx]

**Supplementary table 1.** Primer sequences of various genes in this study.

Table 1. Primer sequences of various genes studied.

| **Name** | **Primer Sequence (5´-3´)** | **Annealing temperature** |
| --- | --- | --- |
| CXCL10- forward | CCAAGTGCTGCCGTCATTTTC | 60℃ |
| CXCL10- reverse | GGCTCGCAGGGATGATTTCAA |  |
| IL-1β- forward | ACAGCAGCACATCAACAAGAG | 60℃ |
| IL-1β- reverse | ATGGGAACGTCACACACCAG |  |
| IL-6- forward | GACAAAGCCAGAGTCCTTCAGAGAGATACAG | 60℃ |
| IL-6- reverse | TTGGATGGTCTTGGTCCTTAGCCAC |  |
| IL-7- forward | TTCCTCCACTGATCCTTGTTCT | 60℃ |
| IL-7- reverse | AGCAGCTTCCTTTGTATCATCAC |  |
| IL-15- forward | AGAGGCCAACTGGATAGATGT | 60℃ |
| IL-15- reverse | AGAGCACGTTTCTTACTGTTTCA |  |
| IL-12p40-forward | TGGTTTGCCATCGTTTTGCTG | 60℃ |
| IL-12p40-reverse | ACAGGTGAGGTTCACTGTTTCT |  |
| TNF-α- forward | TTCTGTCTACTGAACTTCGGGGTGATCGGTCC | 60℃ |
| TNF-α-reverse | GTATGAGATAGCAAATCGGCTGACGGTGTGGG |  |
| IL-10- forward | GCTCTTACTGACTGGCATGAG | 55℃ |
| IL-10-reverse | CGCAGCTCTAGGAGCATGTG |  |
| FOXO3- forward | CTGGGGGAACCTGTCCTATG | 60℃ |
| FOXO3- reverse | TCATTCTGAACGCGCATGAAG |  |
| PD-L1- forward | GCTTCTCAATGTGACCAGCA | 60℃ |
| PD-L1- reverse | GAGGAGGACCGTGGACACTA |  |
| VEGFR2- forward | TTTGGCAAATACAACCCTTCAGA | 60℃ |
| VEGFR2- reverse | GCAGAAGATACTGTCACCACC |  |
| IDO1- forward | CAAAGCAATCCCCACTGTATCC | 60℃ |
| IDO1- reverse | ACAAAGTCACGCATCCTCTTAAA |  |
| IDO2- forward | CCAGAAGGACCGTTGGAAATC | 60℃ |
| IDO2- reverse | ACTGTCACTAGGATGAAGCCC |  |
| HO-1- forward | AAGCCGAGAATGCTGAGTTCA | 60℃ |
| HO-1- reverse | GCCGTGTAGATATGGTACAAGGA |  |
| STAT3- forward | AATATAGCCGATTCCTGCAAGAG | 60℃ |
| STAT3- reverse | TGGCTTCTCAAGATACCTGCTC |  |
| CD86- forward | GAGCTGGTAGTATTTTGGCAGG | 60℃ |
| CD86- reverse | GGCCCAGGTACTTGGCATT |  |
| MHC II- forward | AGCCCCATCACTGTGGAGT | 60℃ |
| MHC II- reverse | GATGCCGCTCAACATCTTGC |  |
| iNOS- forward | ACATCGACCCGTCCACAGTAT | 60℃ |
| iNOS - reverse | CAGAGGGGTAGGCTTGTCTC |  |
| CD206- forward | GCAGGTGGTTTATGGGATGT | 60℃ |
| CD206- reverse | GGGTTCAGGAGTTGTTGTGG |  |
| Arg-1- forward | TTGGGTGGATGCTCACACTG | 60℃ |
| Arg-1 - reverse | GTACACGATGTCTTTGGCAGA |  |
| CCR2- forward | ATCCACGGCATACTATCAACATC | 60℃ |
| CCR2- reverse | CAAGGCTCACCATCATCGTAG |  |
| CSF1R- forward | TGTCATCGAGCCTAGTGGC | 60℃ |
| CSF1R - reverse | CGGGAGATTCAGGGTCCAAG |  |
| TRAILR- forward | CGGGCAGATCACTACACCC | 60℃ |
| TRAILR - reverse | TGTTACTGGAACAAAGACAGCC |  |
| C5aR- forward | ATGGACCCCATAGATAACAGCA | 60℃ |
| C5aR - reverse | GAGTAGATGATAAGGGCTGCAAC |  |
| VISTA- forward | GGAACCCTGCTCCTTGCTATT | 60℃ |
| VISTA- reverse | TTGTAGATGGTCACATCGTGC |  |
| COX-1- forward | GCACGGATAGTAACAACAGGGA | 60℃ |
| COX-1- reverse | GCACGGATAGTAACAACAGGGA |  |
| COX-2- forward | TTCAACACACTCTATCACTGGC | 60℃ |
| COX-2- reverse | AGAAGCGTTTGCGGTACTCAT |  |
| IL-13R- forward | TCAGCCACCTGTGACGAATTT | 60℃ |
| IL-13R - reverse | TGAGAGTGCAATTTGGACTGG |  |
| IL-4R- forward | TCTGCATCCCGTTGTTTTGC | 60℃ |
| IL-4R - reverse | GCACCTGTGCATCCTGAATG |  |
| GAPDH- forward | AGGTCGGTGTGAACGGATTTG | 60℃ |
| GAPDH- reverse | GGGGTCGTTGATGGCAACA |  |
