# Supplementary figures and images for: Cryo-thermal therapy induces macrophage polarization for durable anti-tumor immunity
Source: Cell Death Dis. 2019 Mar 4;10(3):216. doi: 10.1038/s41419-019-1459-7 (PMC6399266; doi:10.1038/s41419-019-1459-7)

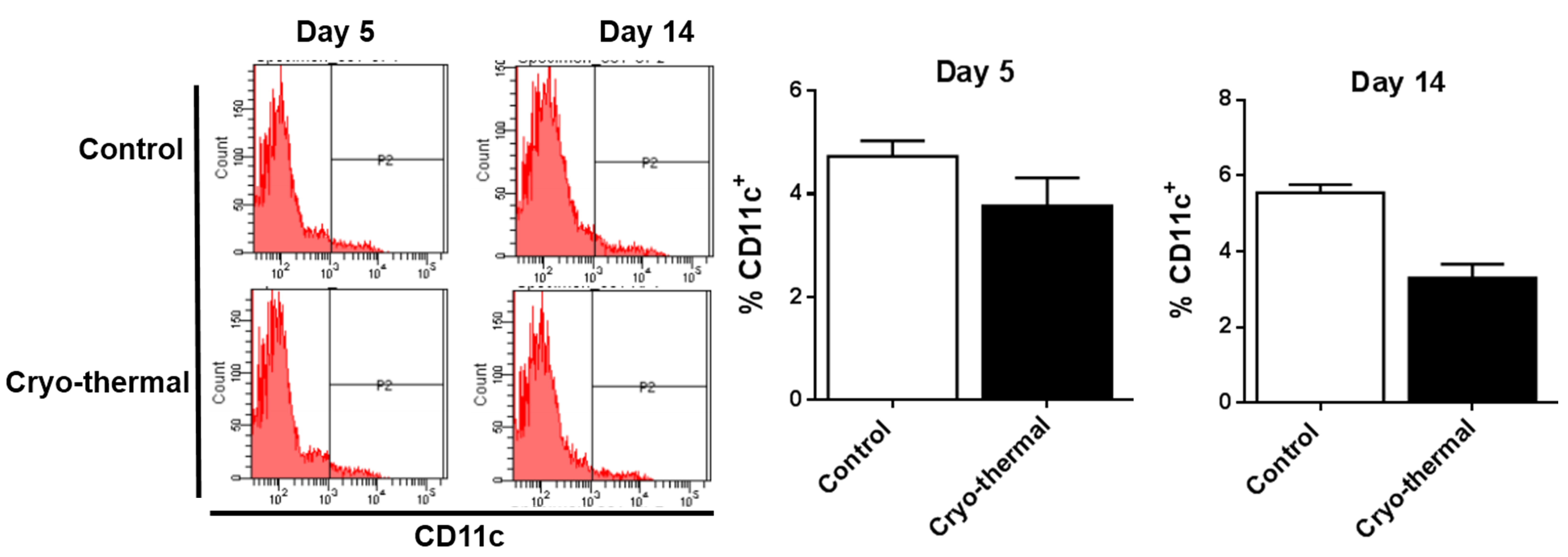

Supplement: Supplementary file 2 — Supplemental Figure 1. The change of splenic CD11c+ DCs [file 41419_2019_1459_MOESM2_ESM.tif]

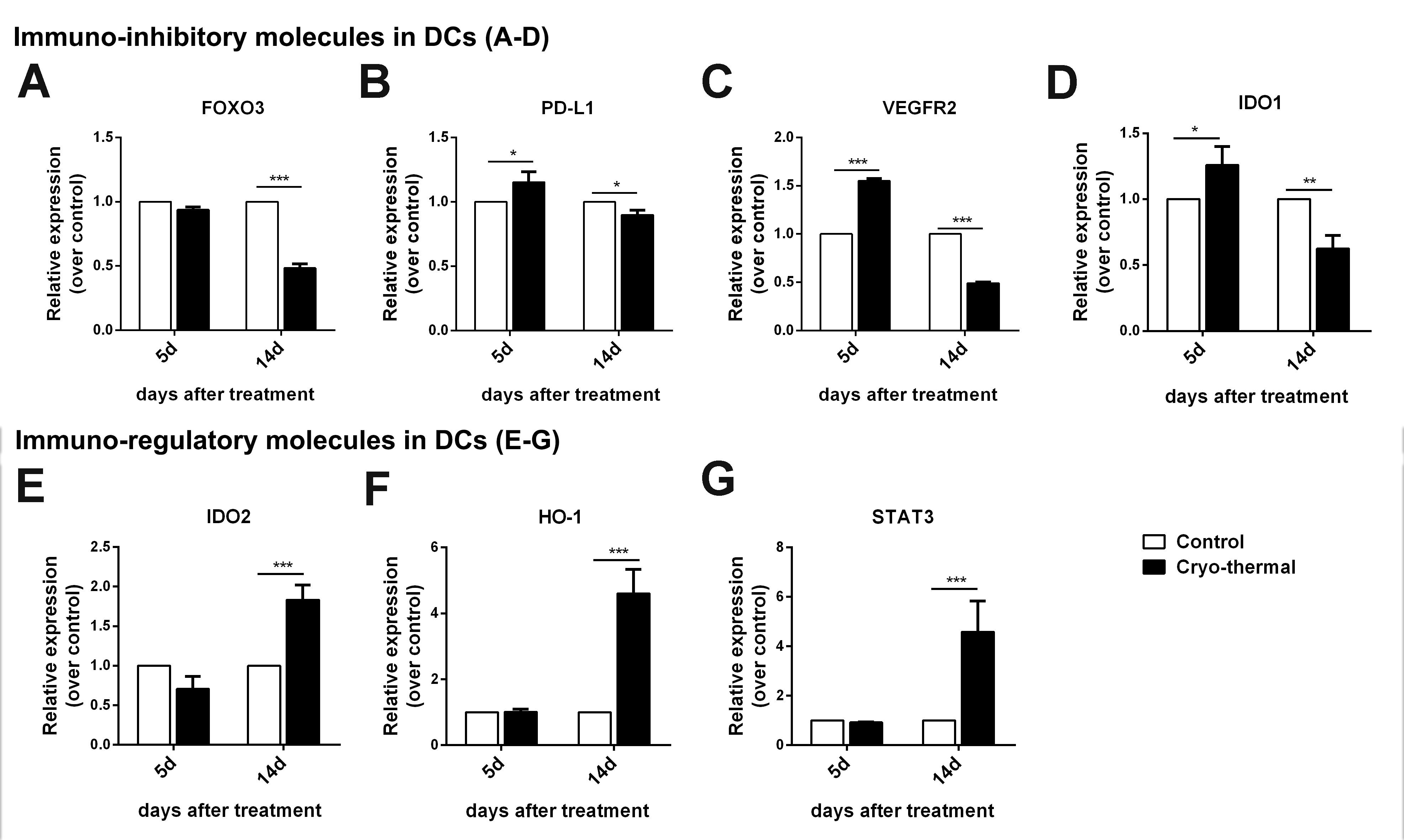

Supplement: Supplementary file 3 — Supplemental Figure 2. The expression of immunoinhibitory or regulatory molecules on splenic CD11c+ DCs after cryo-thermal therapy [file 41419_2019_1459_MOESM3_ESM.tif]

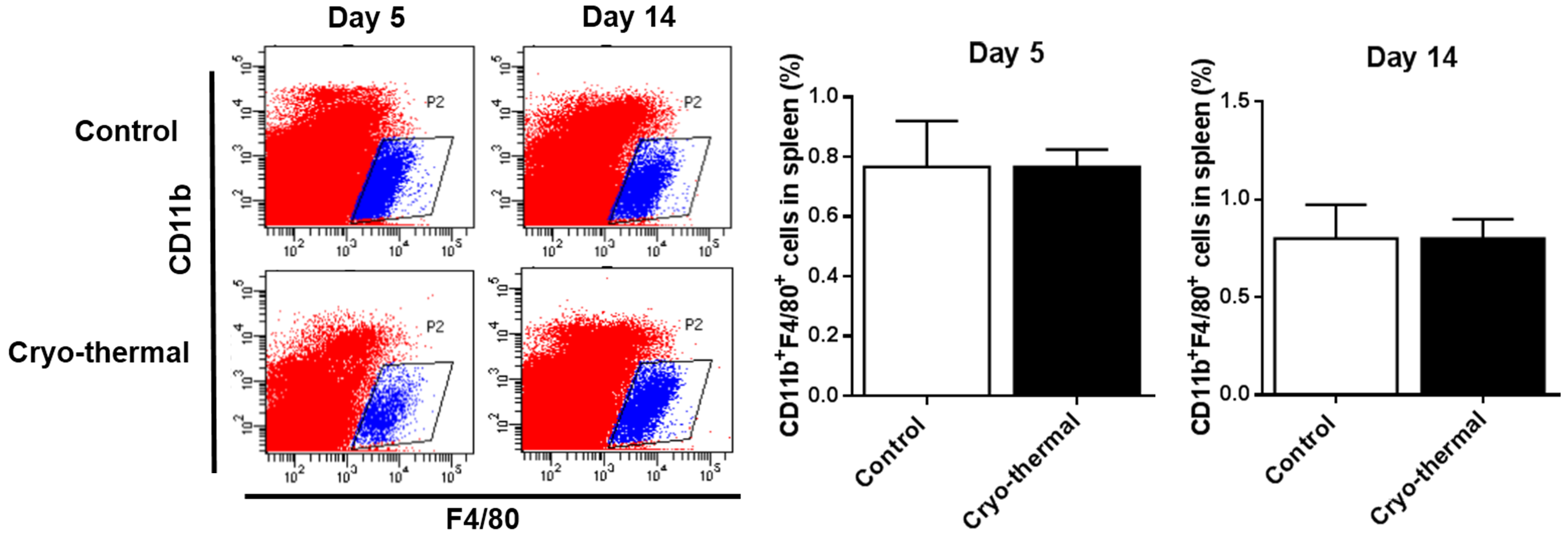

Supplement: Supplementary file 4 — Supplemental Figure 3. The change of splenic CD11b+F4/80+ macrophages [file 41419_2019_1459_MOESM4_ESM.tif]

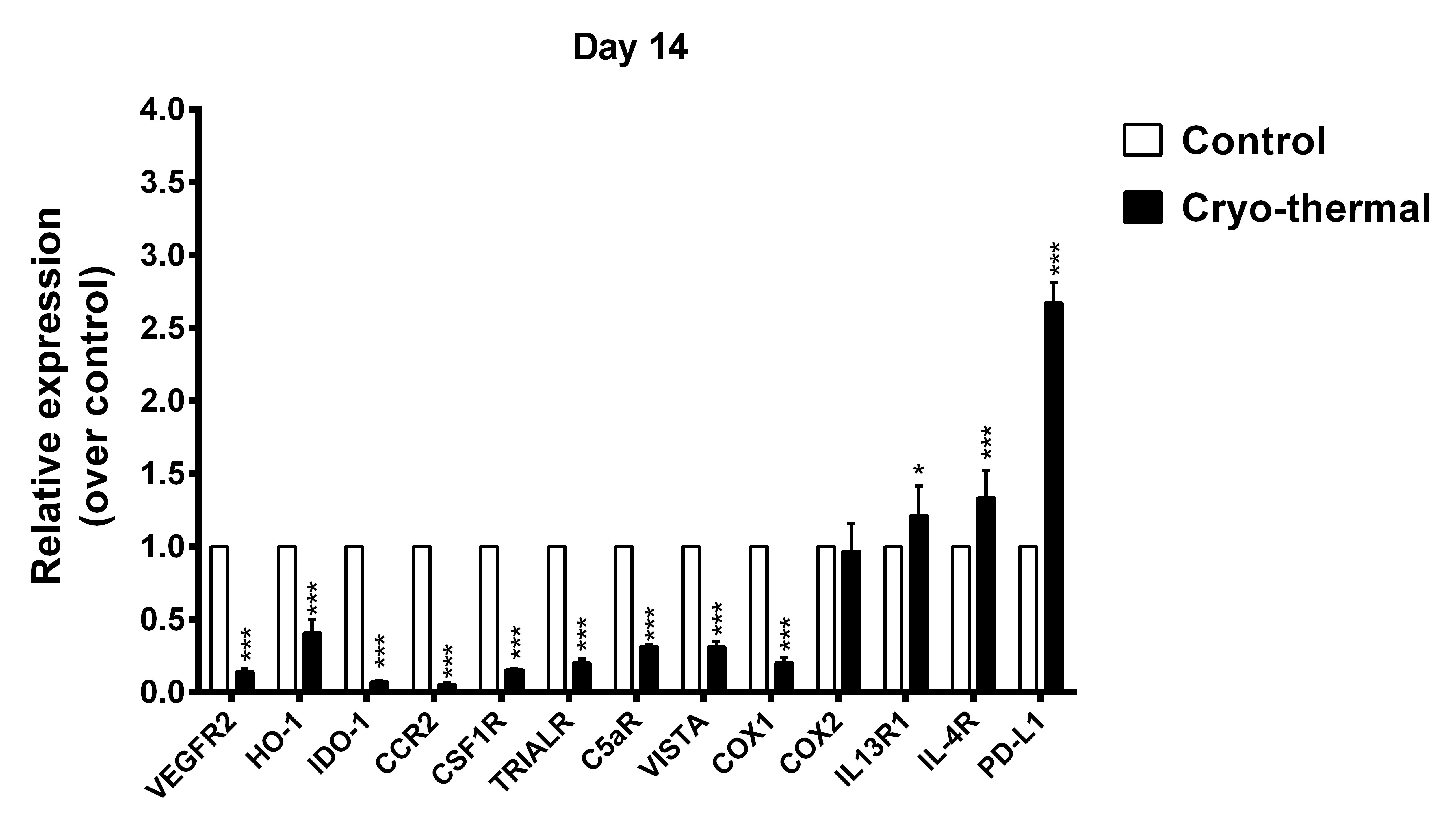

Supplement: Supplementary file 5 — Supplemental Figure 4. The expression of immunoinhibitory or regulatory molecules on splenic CD68+ macrophages after cryo-thermal therapy [file 41419_2019_1459_MOESM5_ESM.tif]

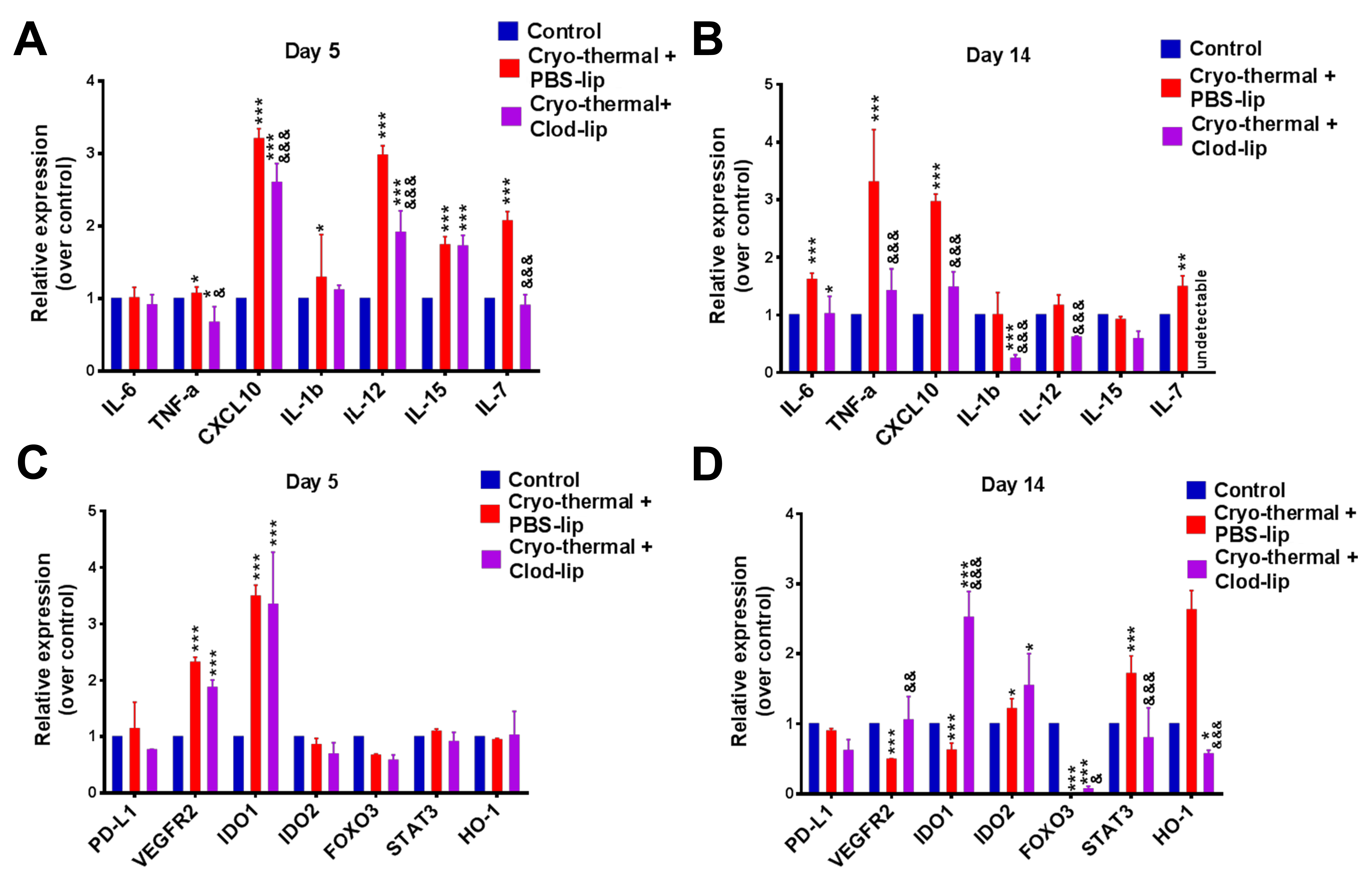

Supplement: Supplementary file 6 — Supplemental Figure 5. The expression of pro-inflammatory cytokines, immunoinhibitory or regulatory molecules on splenic CD11c+ DCs after cryo-thermal therapy plus Clod-lip treatment [file 41419_2019_1459_MOESM6_ESM.tif]

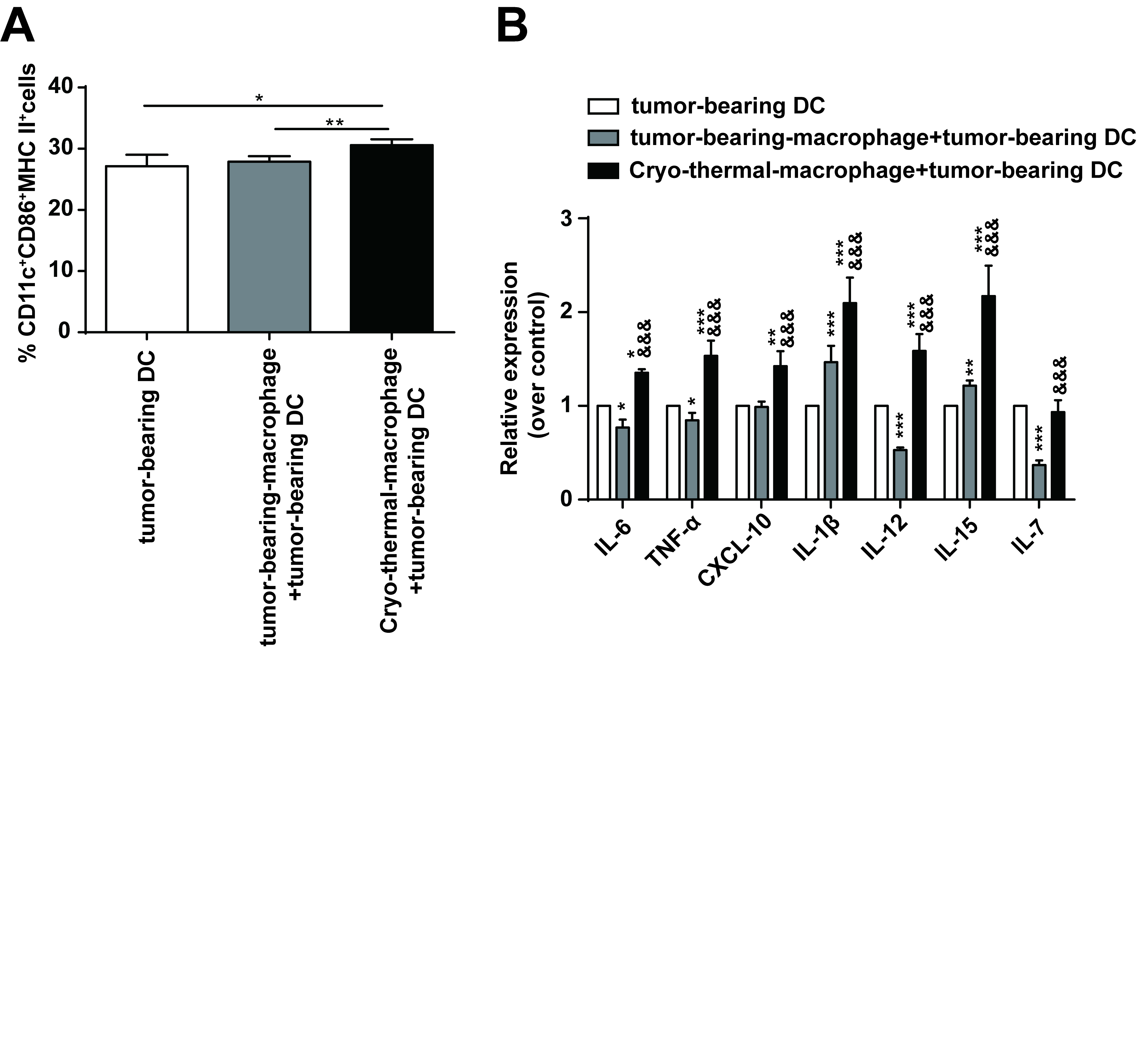

Supplement: Supplementary file 7 — Supplemental Figure 6. Cryo-thermal-re-educated splenic macrophages restored the phenotypic maturation of tumor-bearing DCs in vitro [file 41419_2019_1459_MOESM7_ESM.tif]

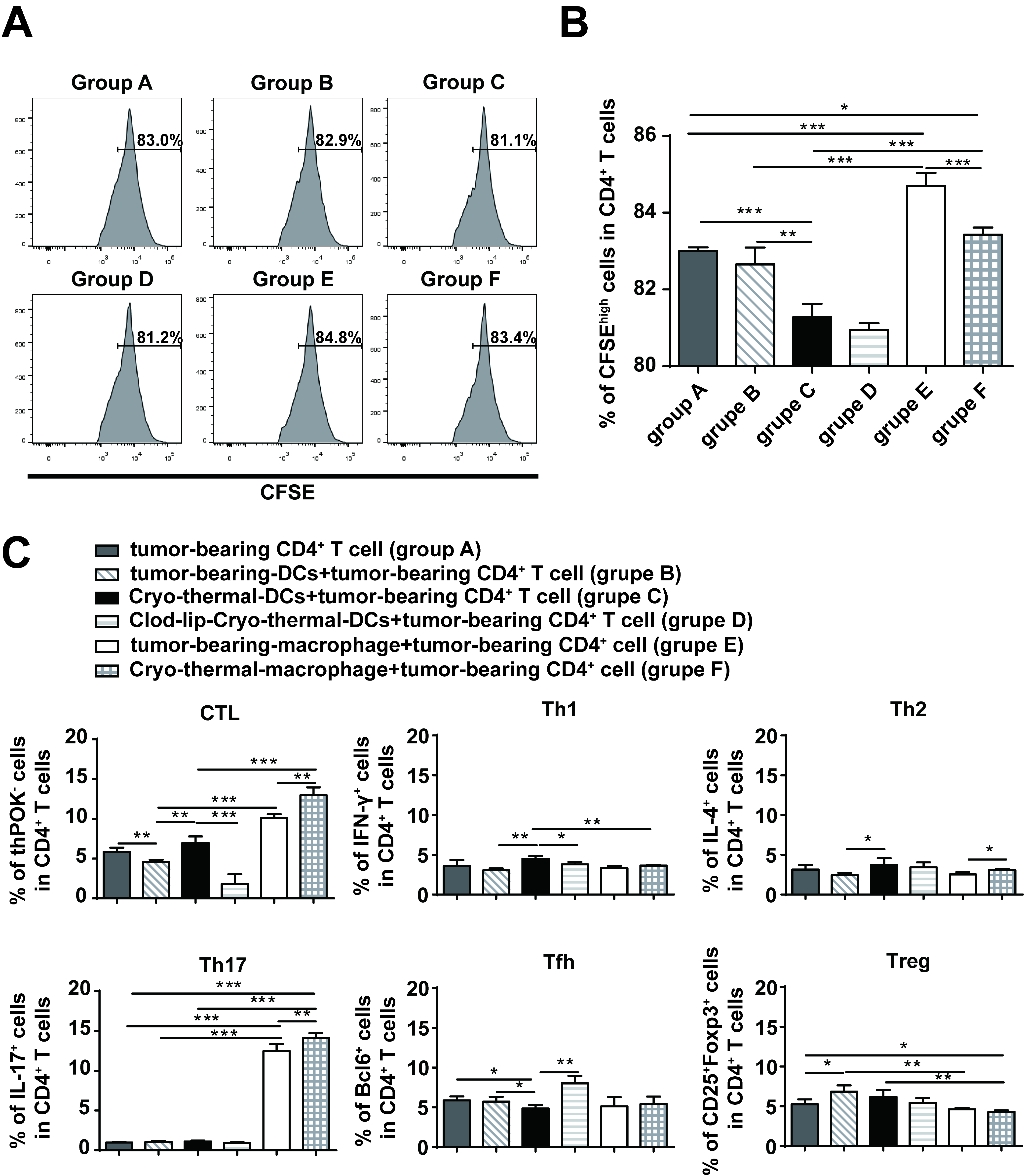

Supplement: Supplementary file 8 — Supplemental Figure 7. Cryo-thermal-re-educated splenic macrophages were required for promotion of functional polarized CD4+ T cells in vitro [file 41419_2019_1459_MOESM8_ESM.tif]

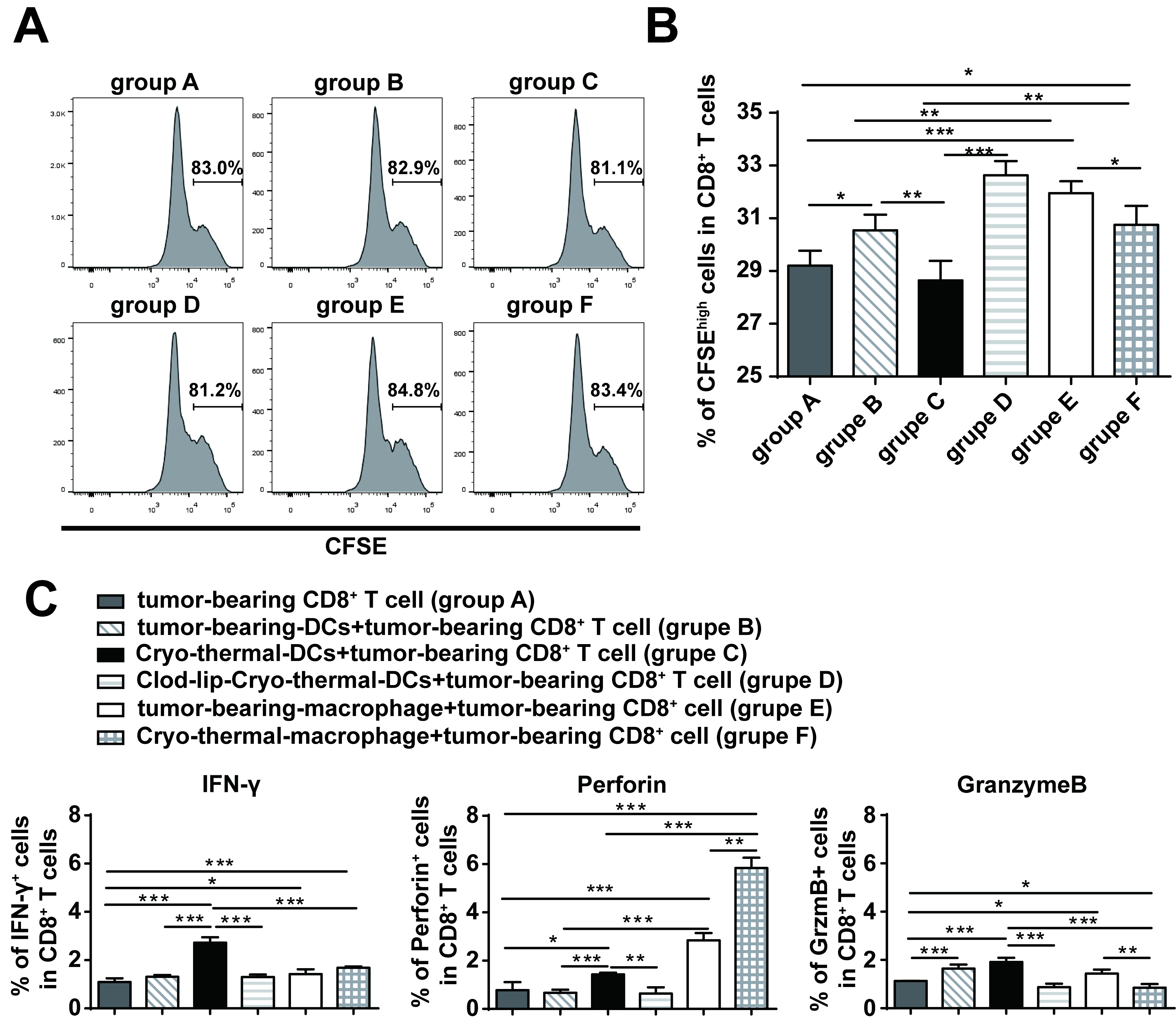

Supplement: Supplementary file 9 — Supplemental Figure 8. Cryo-thermal-induced macrophage polarization to the M1 phenotype was required for promoting cytotoxic CD8+ T cells in vitro [file 41419_2019_1459_MOESM9_ESM.tif]

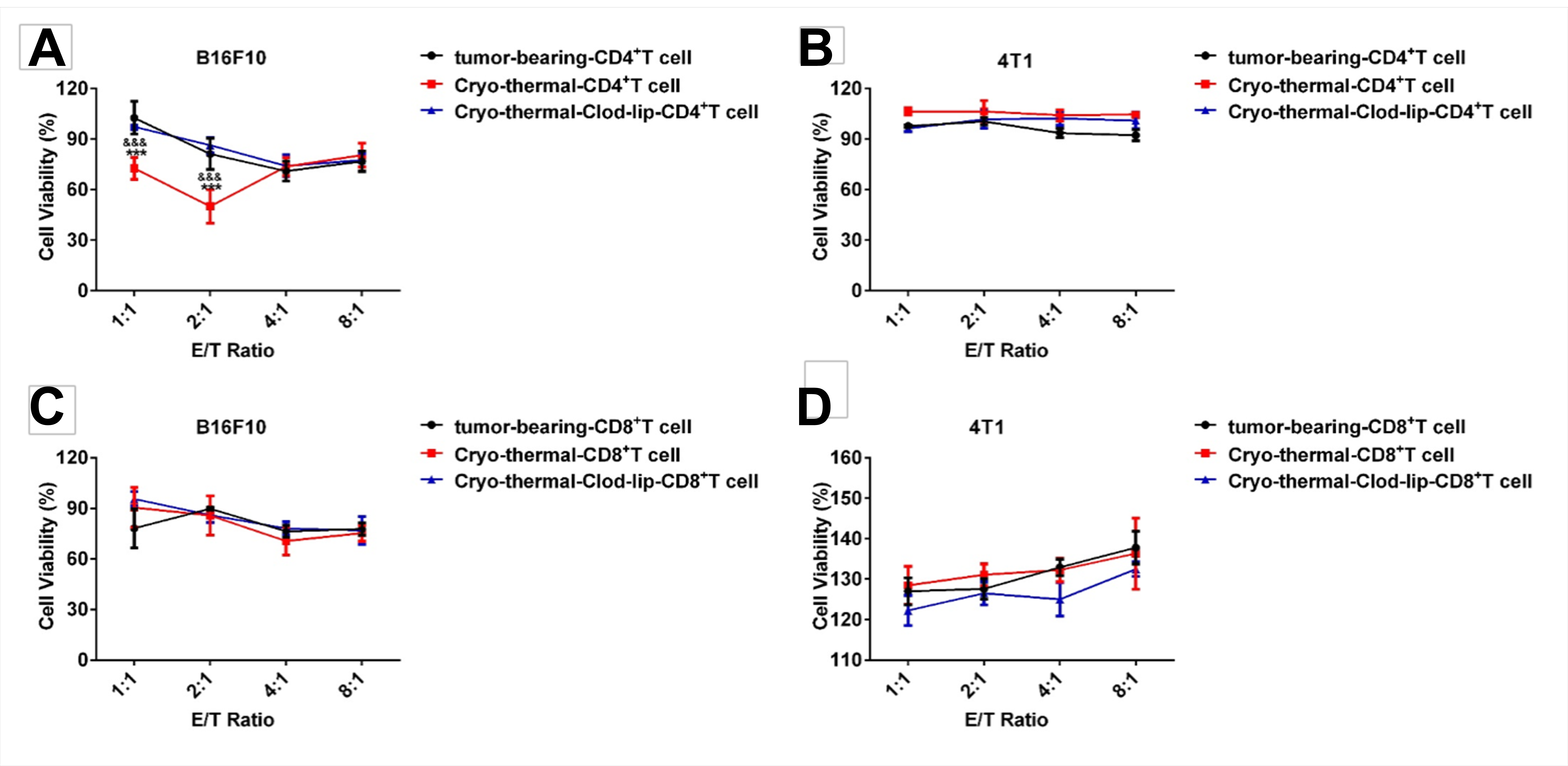

Supplement: Supplementary file 10 — Supplemental Figure 9. Effect of specific cytotoxic T cells (CTLs) mediated by CD4+ T cells after cryo-thermal therapy [file 41419_2019_1459_MOESM10_ESM.tif]
