# Supplementary material for: Cryo-thermal therapy induces macrophage polarization for durable anti-tumor immunity
Source: Cell Death Dis. 2019 Mar 4;10(3):216. doi: 10.1038/s41419-019-1459-7 (PMC6399266; doi:10.1038/s41419-019-1459-7)
Supplement: Supplementary file 11 — Supplemental figure legends [file 41419_2019_1459_MOESM11_ESM.docx]

**Supplementary Figure legends**

**Supplementary Figure 1** The change of splenic CD11c^+^ DCs. Flow cytometry analysis of splenic CD11c^+^ DCs from the treated mice by cryo-thermal therapy and untreated tumor-bearing mice.

**Supplementary Figure 2** The expression of immunoinhibitory or regulatory molecules on splenic CD11c^+^ DCs after cryo-thermal therapy. Splenic CD11c^+^DCs were harvested 5 or 14 days after the treatment to isolate total RNA. PCR analysis was performed to assess the level of *FOXO3* (**A**), *PD-L1*(**B**), *VEGFR2*(**C**), *IDO1*(**D**), *IDO2*(**E**), *HO-1*(**F**) and *STAT3*(**G**). Data was shown as mean ± SD. Data for bar graphs was calculated using two-way ANOVA. * p＜0.05; ** p＜0.01; *** p＜0.001.

**Supplementary Figure 3** The change of splenic CD11b^+^F4/80^+^ macrophages. Flow cytometry analysis of splenic CD11b^+^F4/80^+^macrophages in the treated mice by cryo-thermal therapy and untreated tumor-bearing mice.

**Supplementary Figure 4** The expression of immunoinhibitory or regulatory molecules on splenic CD68^+^ macrophages after cryo-thermal therapy. Splenic CD68^+^macrophages were harvested 14 days after the treatment to isolate total RNA. PCR analysis was performed to assess the level of *VEGFR2*, *HO-1*, *IDO1*, *CCR2*, *CSF1R*, *TRIALR*, *C5aR*, *VISTA*, *COX1*, *COX2*, *IL-13R1*, *IL-4R and PD-L1*. Data was shown as mean ± SD. Data for bar graphs was calculated using two-way ANOVA. Each value of ^*^ p＜0.05 or ^**^ p＜0.01 or ^***^ p＜0.001 was considered to be statistically significant compared with control group.

**Supplementary Figure 5** The expression of pro-inflammatory cytokines, immunoinhibitory or regulatory molecules on splenic CD11c^+^ DCs after cryo-thermal therapy plus Clod-lip treatment. Splenic CD11c^+^DCs were harvested 5 or 14 days after the treatment to isolate total RNA. PCR analysis was performed to assess the level of pro-inflammatory cytokines (**A-B**) and immunoinhibitory or regulatory molecules (**C-D**). Data was shown as mean ± SD. Data for bar graphs was calculated using two-way ANOVA. Each value of * p＜0.05 or ** p＜0.01 or *** p＜0.001 was considered to be statistically significant compared with control group, ^&&&^ p＜0.001 was considered to be statistically significant compared with cryo-thermal+PBS-lip group. *IL-12* was refer to *IL-12p40* in the figure.

**Supplementary Figure 6** Cryo-thermal-re-educated splenic macrophages restored the phenotypic maturation of tumor-bearing DCs *in vitro*. The isolated CD68^+^ macrophages from 14 days after cryo-thermal-treated mice or untreated tumor-bearing mice were co-cultured with CD11c^+^ DCs from the tumor-bearing mice at a ratio of 1:1 for 24 h. (**A**) The percentage of CD11c^+^CD86^+^MHC II^+^ DCs were analyzed by flow cytometry. All data was shown as mean ± SD. * p＜0.05 or ** p＜0.01. Data for bar graphs was calculated using student’s t-test. The co-cultured CD11c^+^ DCs were re-isolated for real-time PCR analysis. The cultured CD11c^+^ DCs from the tumor-bearing mice were used as control. (**B**) The expression of inflammatory cytokines (*IL-6*, *TNF-α*, *CXCL10*, *IL-1β* , *IL-12p40*, *IL-15 and IL-7*) were shown. Data was shown as mean ± SD. ^*^ p＜0.05 or ^**^ p＜0.01 or ^***^ p＜0.001 was considered to be statistically significant compared with that in tumor-bearing DC group. ^&&&^p＜0.001 was considered to be statistically significant compared with that in tumor-bearing macrophage + tumor-bearing DC group. Data for bar graphs was calculated using two-way ANOVA. *IL-12* was refer to *IL-12p40* in the figure.

**Supplementary Figure 7** Cryo-thermal-re-educated splenic macrophages were required for promotion of functional polarized CD4^+^ T cells *in vitro*. (**A-B**) The proliferation activity of CD3^+^CD4^+^ T cells after labeled with CFSE in co-cultures were detected by flow cytometry. Data was shown as mean ± SD. Data for bar graphs were calculated using student’s t-test. ^*^ p＜0.05 or ^**^ p＜0.01 or ^***^ p＜0.001. (**C**) The co-cultured CD4^+^ T cells were purified for flow cytometry assay. The level of thPOK (for CD4-CTL cells); IFN-γ (for Th1 cells); IL-4 (for Th2 cells); IL-17 (for Th17 cells); Bcl-6 (for Tfh cells) and FoxP3 (for Treg cells) in CD4^+^ T cells were examined by flow cytometry. Data was shown as mean ± SD. Data for bar graphs was calculated using student’s t-test. ^*^ p＜0.05 or ^**^ p＜0.01 or ^***^ p＜0.001.

**Supplementary Figure 8** Cryo-thermal-induced macrophage polarization to the M1 phenotype was required for promoting cytotoxic CD8^+^ T cells *in vitro*. (**A-B)** The proliferation activity of CD3^+^CD8^+^ T cells after labeled with CFSE in co-cultures were detected by flow cytometry. Data was shown as mean ± SD. Data for bar graphs were calculated using student’s t-test. ^*^ p＜0.05 or ^**^ p＜0.01 or ^***^ p＜0.001. (**C**) The level of IFN-γ, perforin and granzyme B in CD8^+^ T cells were examined by flow cytometry. Data was shown as mean ± SD. Data for bar graphs was calculated using student’s t-test. ^*^ p＜0.05 or ^**^ p＜0.01 or ^***^ p＜0.001.

**Supplementary Figure 9** Effect of specific cytotoxic T cells (CTLs) mediated by CD4^+^ T cells after cryo-thermal therapy. Splenic CD4^+^ or CD8^+^ T cells from Cryo-thermal, Cryo-thermal-Clod-lip, and tumor-bearing groups were purified and mixed with B16F10 or 4T1 cells at the indicated ratios, and the cell viability (%) was determined. (**A**) The B16F10 tumor cell viability (%) in co-cultured tumor-bearing-CD4^+^ T cells, Cryo-thermal-CD4^+^ T cells, and Cryo-thermal-Clod-lip-CD4^+^ T cells. (**B**) The B16F10 tumor cell viability (%) in the tumor-bearing-CD8^+^T cell group, Cryo-thermal-CD8^+^ T cell group, and Cryo-thermal-Clod-lip-CD8^+^ T cell group. (**C**) The 4T1 tumor cell viability (%) in tumor-bearing-CD4^+^ T cell group, Cryo-thermal-CD4^+^ T cell group, and Cryo-thermal-Clod-lip-CD4^+^ T cell group. (**D**) The 4T1 tumor cell viability (%) in the tumor-bearing-CD8^+^ T cell group, Cryo-thermal-CD8^+^ T cell group, and Cryo-thermal-Clod-lip-CD8^+^ T cell group. Data is shown as mean ± SD. ^***^ p < 0.001, Cryo-thermal-CD4^+^ T cell group compared with tumor-bearing-CD4^+^T cell group; ^&&&^ p < 0.001, Cryo-thermal-CD4^+^T cell group compared with Cryo-thermal-Clod-lip-CD4^+^T cell group. Data for bar graphs was calculated using one-way ANOVA.
